# Glycaemic and Cardiometabolic Outcomes of Empagliflozin Versus Sitagliptin Added to Metformin in T2DM: Insights From a Systematic Review and Meta‐Analysis

**DOI:** 10.1002/edm2.70238

**Published:** 2026-05-12

**Authors:** Saad Ashraf, Muhammad Burhan, Sara Sarwar, Ahila Ali, Aiza Ahsan, Shahzad Ashraf, Hammad Javaid, Hamza Irfan, Muhammad Hamza Naseer Awan, Ajeet Singh, Biruk Demisse Ayalew

**Affiliations:** ^1^ Department of Medicine Dow University of Health Sciences Karachi Pakistan; ^2^ Department of Medicine King Edward Medical University Lahore Pakistan; ^3^ Department of Medicine Shaikh Khalifa Bin Zayed Al Nahyan Medical and Dental College Lahore Pakistan; ^4^ Department of Internal Medicine St. Paul's Hospital Millennium Medical College Addis Ababa Ethiopia

**Keywords:** cardiometabolic outcomes, empagliflozin, meta‐analysis, metformin, sitagliptin, type 2 diabetes

## Abstract

**Background:**

Type 2 diabetes mellitus (T2DM) often requires combination therapy when metformin alone becomes insufficient. Empagliflozin (SGLT2 inhibitor) and sitagliptin (DPP‐4 inhibitor) are commonly used add‐on agents with differing metabolic profiles. This systematic review and meta‐analysis compared their glycaemic, cardiometabolic and safety outcomes when added to metformin.

**Methods:**

Following PRISMA guidelines, PubMed, Embase, Cochrane, Scopus and ClinicalTrials.gov were searched from inception to September 2025. Randomized trials and observational studies comparing empagliflozin + metformin versus sitagliptin + metformin in adults with T2DM were included. Primary outcomes were changes in HbA1c, body weight, fasting glucose, lipid profile and blood pressure. Safety outcomes included urinary tract infections, genital infections, gastrointestinal disturbances and rash. Risk of bias was assessed using RoB 2.0 and the Newcastle–Ottawa Scale. Random‐effects models were used for meta‐analyses, and meta‐regression explored the impact of empagliflozin dose.

**Results:**

Eleven studies met eligibility criteria. Empagliflozin produced greater reductions in HbA1c, body weight, fasting glucose and systolic blood pressure compared with sitagliptin. Lipid changes were modest and inconsistent. Rates of urinary infections, gastrointestinal symptoms and rash were comparable between groups, whereas genital infections were significantly higher with empagliflozin. Rare but serious adverse events associated with SGLT2 inhibitors, including Fournier's gangrene and lower limb amputations, were not reported in the included trials. Meta‐regression showed no meaningful dose–response relationship for glycaemic or weight outcomes.

**Conclusions:**

In patients with T2DM on metformin, empagliflozin offers superior glycaemic and cardiometabolic benefits compared with sitagliptin, with an increase in genital infections. Both therapies are well tolerated, supporting empagliflozin as an effective metabolic add‐on option.

**Trial Registration:**

PROSPERO ID: CRD420251152360

## Graphical abstract

Empagliflozin added to metformin provides greater HbA1c reduction, weight loss and systolic blood pressure improvement compared with sitagliptin. However, this benefit is accompanied by a higher risk of genital infections, while other safety outcomes remain largely comparable between therapies.

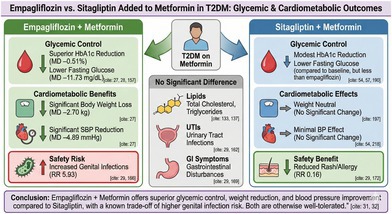

## 1 Introduction

Type 2 diabetes mellitus (T2DM) represents a profound global health challenge, characterized as a chronic metabolic disorder leading to significant morbidity and mortality. The International Diabetes Federation (IDF) reports that one in every 11 adults aged 20–79 years is affected by diabetes, with a global prevalence of 8.8% [1]. Current estimates indicate that approximately 463 million people worldwide are living with diabetes, a figure projected to rise to 629 million by 2045 [1, 2]. This epidemic is more severe in some areas than others; in India, for example, the prevalence is estimated to be 11% and is predicted to rise to 15% by 2030 [3]. Moreover, more than 95% of all instances of diabetes are type 2 diabetes, which is a major cause of death due to its macro‐ and microvascular complications [2, 4].

The management of T2DM is complex, aimed at achieving and maintaining optimal glycaemic control to prevent long‐term complications. Metformin, a biguanide insulin sensitizer, is universally endorsed as the first‐line pharmacological agent following lifestyle modifications [3, 4]. However, because β‐cell function decline in type 2 diabetes is gradual, metformin monotherapy frequently becomes insufficient over time for most patients to maintain their glycaemic targets [5]. This progressive β‐cell dysfunction necessitates the timely addition of complementary anti‐hyperglycaemic agents. According to current clinical guidelines, if glycated haemoglobin (HbA1c) levels are between 7.5% and 9.0%, or if 3 months of monotherapy yields no improvement, dual therapy with metformin plus a second agent should be started. The choice of the safest and most effective second‐line add‐on therapy continues to be a crucial and evolving clinical practice question [3, 6].

Dipeptidyl peptidase‐4 (DPP‐4) and sodium‐glucose cotransporter‐2 (SGLT2) inhibitors are the two main medication groups utilized in addition to metformin; they work through distinct mechanisms of action. The potent SGLT2 inhibitor empagliflozin increases urine glucose excretion via decreasing renal glucose reabsorption in the proximal tubule, which occurs independently of insulin [2, 5]. This mechanism not only lowers plasma glucose but also promotes weight loss due to caloric wasting and offers blood pressure‐lowering benefits [1, 7]. Furthermore, empagliflozin has demonstrated significant cardio‐renal protective effects, including a reduction in the risk of cardiovascular mortality [1, 7]. Landmark evidence from the EMPA‐REG OUTCOME trial reported a 38% reduction in cardiovascular death and a 35% lower risk of hospitalization for heart failure, underscoring its cardioprotective potential [8].

Conversely, sitagliptin is a competitive DPP‐4 inhibitor that increases the activity of endogenous incretin hormones such as glucose‐dependent insulinotropic polypeptide (GIP) and glucagon‐like peptide‐1 (GLP‐1). Sitagliptin inhibits the release of glucagon from the pancreas and promotes glucose‐dependent insulin secretion by blocking the breakdown of these hormones. Although DPP‐4 inhibitors are good at reducing HbA1c, their cardiovascular benefits are regarded as neutral, as recent meta‐analyses and the TECOS trial have found no discernible difference in cardiovascular events when compared to a placebo [1, 3, 9, 10].

The comparative profile of the glycaemic and cardiometabolic effects of empagliflozin and sitagliptin when combined to metformin is still being studied clinically, despite their proven effectiveness. Among these classes, empagliflozin and sitagliptin were selected as the primary focus because they represent the most widely utilized and extensively researched agents within the SGLT2i and DPP‐4i families, respectively. Findings from a number of studies are essential to summarize. A comparison study conducted in 2024, for instance, found that the combination of empagliflozin and metformin is better than sitagliptin and metformin for preserving glycaemic control, with a noticeably higher decrease in body weight and HbA1c (−1.34% vs. −0.65%) [1, 3, 4]. Furthermore, in comparison to the sitagliptin‐metformin combination, another study verified empagliflozin's better effectiveness in lowering body weight, HbA1c, fasting blood sugar, and improving lipid profiles and blood pressure. Nonetheless, some research, including one study, indicated that the combination of sitagliptin and metformin may provide greater lipid profile benefits by more efficiently lowering levels of low‐density lipoprotein (LDL) and total cholesterol.

A thorough, high‐level evidence synthesis is required in light of this conflicting evidence, as well as differences in study designs, populations and follow‐up durations [1, 2, 11]. This systematic review and meta‐analysis aims to quantitatively consolidate the existing evidence from RCTs and observational studies to provide a definitive comparative assessment of the glycaemic and cardiometabolic outcomes of empagliflozin versus sitagliptin as an add‐on therapy to metformin in patients with T2DM.

## 2 Methods

### 2.1 Protocol Registration

This systematic review and meta‐analysis was performed according to the Preferred Reporting Items for Systematic Reviews and Meta‐Analysis (PRISMA) guidelines and the Cochrane Handbook of Systematic Reviews of Intervention [12]. As per standard recommendations, this review was registered in the (PROSPERO) [13].

### 2.2 Data Sources and Search Strategy

A detailed literature search was conducted across PubMed, Embase, Cochrane, ClinicalTrials.gov and Scopus databases to identify studies that fulfilled the predefined eligibility criteria from inception till September 2025. The search strategy was independently outlined according to the PRISMA standards by two reviewers (H.J. and A.A.). The reference lists of all included studies were meticulously reviewed for additional information. The search was conducted using appropriate keywords and medical subject headings (MeSH) terms such as ‘empagliflozin’; ‘sitagliptin’; ‘metformin’; ‘type 2 diabetes mellitus’ joined using Boolean operators. The literature search was not limited to any language or study design filters to avoid missing potentially relevant studies. The detailed search strategy for each database is provided in Table S1.

### 2.3 Study Selection and Eligibility Criteria

After the detailed search of the above‐mentioned databases and directory, all the articles were exported to Rayyan AI web software for screening. Duplicate articles were removed with the help of software and re‐checked manually. Two reviewers (A.A. and M.H.N.A.) independently screened and excluded articles first based on title and abstract and then the full‐length text of the relevant articles, matching our predefined PICOS framework, were retrieved and carefully examined for inclusion. Discrepancies were resolved by a third independent reviewer (H.J.).

The inclusion criteria were formulated based on PICOS (Population, Interventions, Comparison, Outcomes, Study Design). Studies included in this systematic review and meta‐analysis fulfilled the following criteria: (1) enrolled adult participants aged 18 years or older with a clinically confirmed diagnosis of type 2 diabetes mellitus (T2DM) who were receiving metformin as background therapy; (2) evaluated empagliflozin (any approved dose) as the intervention in combination with metformin; (3) included a comparator group receiving sitagliptin (any approved dose) in combination with metformin; (4) reported at least one efficacy outcome such as change in HbA1c or proportion achieving target HbA1c, and/or cardiometabolic or safety outcomes including changes in body weight, blood pressure, lipid profile, hypoglycaemia, infections, cardiovascular or renal events, or treatment‐related adverse events; (5) adopted a study design comprising randomized controlled trials (RCTs), prospective cohort studies or retrospective comparative studies; (6) were published as full‐text, peer‐reviewed articles in English or with English translation available. The selection was intentionally restricted to these two specific agents to minimize pharmacological heterogeneity within their drug classes and ensure the results reflect the most common clinical pairing added to a metformin background. Studies were excluded if: (1) involved participants younger than 18 years, or with type 1 diabetes mellitus, gestational diabetes or other non–type 2 diabetes conditions; (2) evaluated empagliflozin or sitagliptin as monotherapy or in combination with agents other than metformin, without a clearly defined metformin background; (3) lacked a direct or extractable comparison between empagliflozin plus metformin and sitagliptin plus metformin; (4) did not report any relevant efficacy or cardiometabolic/safety outcomes of interest; (5) were designed as case reports, case series, narrative reviews, editorials, commentaries, letters or conference abstracts without sufficient numerical data; (6) were not published as full‐text, peer‐reviewed articles in English or with English translation available.

### 2.4 Data Extraction and Outcome Measures

Relevant information including study characteristics, patient demographics, intervention details, author identification and outcomes of this meta‐analysis including: change in HbA1c, change in body weight, change in total cholesterol, change in triglycerides, change in HDL, change in LDL, change in systolic blood pressure, change in diastolic blood pressure, change in fasting blood glucose, UTI, genital infections, GI disturbances and rash/allergy were extracted and recorded in a standardized form in Excel sheets by two authors (S.A. and A.A.). Any disagreements or inconsistencies were resolved through mutual discussion with a third reviewer (H.J.).

### 2.5 Quality Assessment

Two independent reviewers (S.A. and A.A.) conducted the risk of bias and quality assessment, with a third investigator (S.A.) invited to resolve any discrepancies. The Newcastle–Ottawa Scale (NOS) was used to evaluate the quality of cohorts [14]. Each study received a score out of nine points, with scores of ≥ 7 indicating a low risk of bias, scores between 4 and 6 indicating a moderate risk, and scores of ≤ 3 indicating a high risk of bias. The risk of bias for the RCTs was assessed using the Cochrane Risk of Bias 2.0 (RoB 2) tool [15].

### 2.6 Statistical Analysis

All statistical analyses were performed using R studio (4.5.1) where ‘metacont’ was applied for continuous outcomes and ‘metabin’ for dichotomous outcomes. A *p*‐value of < 0.05 was considered statistically significant. Meta‐regression analysis was also conducted to explore the impact of empagliflozin dose as a potential moderator on treatment outcomes. Regression models estimated the slope and 95% confidence intervals with *p*‐values < 0.05 showing significant association. The proportion of inter‐study variability explained with *R* ^2^ was also reported.

Risk ratios (RRs) were calculated for dichotomous outcomes, while mean differences (MDs) were computed for continuous outcomes. All results were reported with 95% confidence intervals (CIs). Statistical heterogeneity was assessed using the *I* ^2^ statistic, with thresholds of < 20%, 25%–50% and > 50% indicating low, moderate and high heterogeneity, respectively. For datasets with fewer than 10 studies, publication bias was evaluated using a DOI plot and the Luis Furuya‐Kanamori (LFK) index, implemented in R version 4.5.1. For 10 or more studies, Egger's test for funnel plot asymmetry was performed to evaluate publication bias [16].

## 3 Results

### 3.1 Study Selection

**FIGURE 1:**
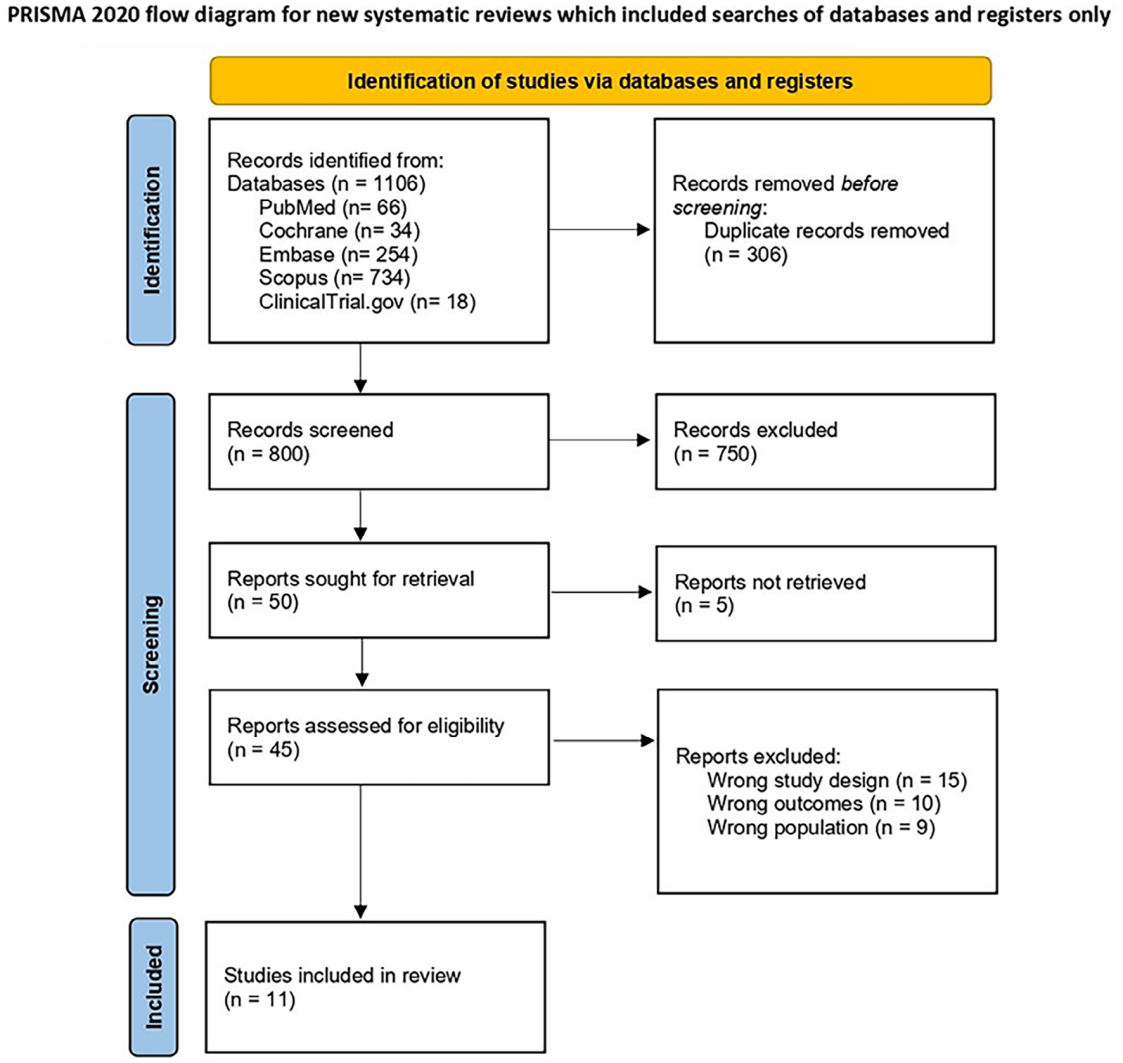
PRISMA flow diagram of study selection.

A comprehensive search across various electronic databases initially identified 1106 records. After the removal of 306 duplicate records, 800 unique articles remained. A preliminary screening of titles and abstracts was conducted based on predefined exclusion criteria, leading to the elimination of 750 studies. Subsequently, 50 studies were sought for retrieval, but 5 could not be accessed. A rigorous full‐text evaluation was then conducted on 45 studies by two independent reviewers, applying both inclusion and exclusion criteria. This process reduced the number of relevant studies to 11 studies, which met all requirements of inclusion. The PRISMA flow chart outlines the systematic screening process as shown in Figure 1.

### 3.2 Study and Patient Characteristics

A total of 11 studies were included in the meta‐analysis, with sample sizes for individual treatment arms ranging from 39 to 166 participants. The studies compared the effects of empagliflozin plus metformin versus sitagliptin plus metformin in adults with type 2 diabetes. The mean age of participants across studies ranged from approximately 41–60 years, with most studies reporting mean ages in the 50s. The proportion of male participants varied widely, from approximately 35%–95%. Most studies enrolled patients with established diabetes; mean diabetes duration, when reported, ranged from approximately 3–5 years.

Baseline body mass index (BMI) values, where available, mostly indicated that patients were overweight or obese, with means commonly in the 28–40 kg/m^2^ range. Baseline HbA1c values were generally in the 7.5%–8.5% range and mean fasting plasma glucose (FPG) levels ranged from about 150–250 mg/dL. Baseline systolic blood pressure (SBP) values, when reported, varied between 120 and 165 mmHg. Study characteristics and baseline clinical parameters are summarized in Table 1.

**TABLE 1:** Baseline characteristics of included studies.

Study ID	Treatment group	*n*	Age (years, mean ± SD)	Male (%)	Duration of diabetes (years, mean ± SD)	Body weight (kg, mean ± SD)	BMI (kg/m^2^, mean ± SD)	SBP (mmHg, mean ± SD)	DBP (mmHg, mean ± SD)	HbA1c (% mean ± SD)	FPG (mg/dL, mean ± SD)	Total cholesterol (mg/dL, mean ± SD)
Ferrannini et al. (2013)	Empagliflozin + Metformin	166	60.0 ± 13.0	50	NR	89.6 ± 15.0	30.2 ± 4.0	134.2 ± 14.6	80.3 ± 8.6	7.9 ± 0.7	175.9 ± 37.7	191.9 ± 16.0
	Sitagliptin + Metformin	166	60.0 ± 13.0	47	NR	89.5 ± 16.2	30.3 ± 4.0	134.3 ± 14.5	80.8 ± 9.6	7.9 ± 0.8	178.3 ± 39.2	195.4 ± 13.8
Haitham et al. (2023)	Empagliflozin + Metformin	75	53.4 ± 10.2	53.3	NR	93.3 ± 18.0	NR	136.7 ± 17.8	85.6 ± 10.8	8.3 ± 0.8	164.6 ± 45.2	207.0 ± 38.8
	Sitagliptin + Metformin	82	53.5 ± 8.7	40.2	NR	91.1 ± 15.4	NR	134.5 ± 16.3	83.0 ± 13.5	8.5 ± 1.0	184.2 ± 64.4	203.1 ± 39.2
Harsh et al. (2024)	Empagliflozin + Metformin	150	41.2 ± 4.1	56.7	3.1 ± 0.9	NR	NR	NR	NR	NR	NR	NR
	Sitagliptin + Metformin	150	43.2 ± 3.2	58.7	3.5 ± 0.9	NR	NR	NR	NR	NR	NR	NR
Ibrar et al. (2022)	Empagliflozin + Metformin	44	44.7 ± 10.7	54.5	NR	NR	NR	NR	NR	8.2 ± 0.4	NR	NR
	Sitagliptin + Metformin	88	50.6 ± 10.5	48.8	NR	NR	NR	NR	NR	8.1 ± 0.4	NR	NR
Javed et al. (2025)	Empagliflozin + Metformin	140	50.6 ± 6.2	58.6	NR	92.5 ± 13.5	29.4 ± 4.2	132.0 ± 15.5	75.0 ± 8.5	8.4 ± 1.9	240.5 ± 60.5	188.0 ± 20.5
	Sitagliptin + Metformin	140	49.3 ± 8.5	53.6	NR	89.0 ± 16.6	28.5 ± 5.6	125.0 ± 18.5	80.0 ± 6.5	8.5 ± 1.5	255.0 ± 46.6	195.0 ± 18.5
Mazhar et al. (2023)	Empagliflozin + Metformin	63	51.8 ± 6.3	95.2	4.3 ± 1.4	NR	40.1 ± 1.1	164.8 ± 12.1	NR	NR	NR	195.4 ± 13.8
	Sitagliptin + Metformin	63	55.2 ± 6.2	68.3	4.3 ± 1.3	NR	40.0 ± 1.5	156.9 ± 13.0	NR	NR	NR	191.9 ± 16.0
Muaz et al. (2022)	Empagliflozin + Metformin	63	55.2 ± 6.3	68.3	4.3 ± 1.3	71.7 ± 6.8	NR	NR	NR	8.7 ± 0.4	NR	NR
	Sitagliptin + Metformin	63	51.8 ± 6.3	95.2	4.4 ± 1.4	67.4 ± 6.8	NR	NR	NR	8.9 ± 0.4	NR	NR
Naimeh et al. (2025)	Empagliflozin + Metformin	46	53.7 ± 8.4	52.1	NR	78.2 ± 8.0	28.4 ± 6.3	132.4 ± 16.3	80.5 ± 10.8	7.9 ± 0.6	153.2 ± 32.3	NR
	Sitagliptin + Metformin	45	54.0 ± 6.9	42.2	NR	77.1 ± 6.3	28.8 ± 5.3	129.4 ± 18.7	78.7 ± 10.7	7.5 ± 0.5	156.9 ± 15.1	NR
Saman et al. (2024)	Empagliflozin + Metformin	40	59.4 ± 8.7	35	5.4 ± 2.5	87.4 ± 13.4	29.6 ± 1.7	126.9 ± 21.7	NR	8.4 ± 0.7	188.3 ± 31.7	203.0 ± 29.5
	Sitagliptin + Metformin	39	56.8 ± 9.8	43.6	4.9 ± 2.3	83.5 ± 11.3	29.2 ± 1.7	119.9 ± 23.6	NR	8.3 ± 0.8	181.2 ± 25.8	191.7 ± 30.7
Hadeeqa et al. (2025)	Empagliflozin + Metformin	56	NR	53.6	NR	NR	NR	NR	NR	8.09 ± 0.42	NR	NR
	Sitagliptin + Metformin	56	NR	58.9	NR	NR	NR	NR	NR	8.18 ± 0.41	NR	NR

Abbreviations: BMI, body mass index; DBP, diastolic blood pressure; FPG, fasting plasma glucose; HbA1c, glycated haemoglobin; *n*, number of participants; NR, not reported; SBP, systolic blood pressure; SD, standard deviation; TC, total cholesterol.

### 3.3 Quality Assessment of Included Studies

Quality assessment of the included studies revealed that the majority of the randomized controlled trials were generally of low risk or some concerns, with most demonstrating robust randomization, objective outcome measures and adequate follow‐up, though open‐label designs or unreported blinding were common minor limitations in several studies as shown in Figure S1. The two observational studies both scored high on the Newcastle–Ottawa Scale, with each receiving 8 out of 9 stars for strong cohort selection, comparability, outcome assessment and follow‐up; though, as with all non‐randomized research, some potential for residual confounding remains as shown in Table S2. Overall, the risk of significant bias across all included studies is low to moderate.

### 3.4 Efficacy Outcomes

#### 3.4.1 Change in HbA1c (%)

**FIGURE 2:**
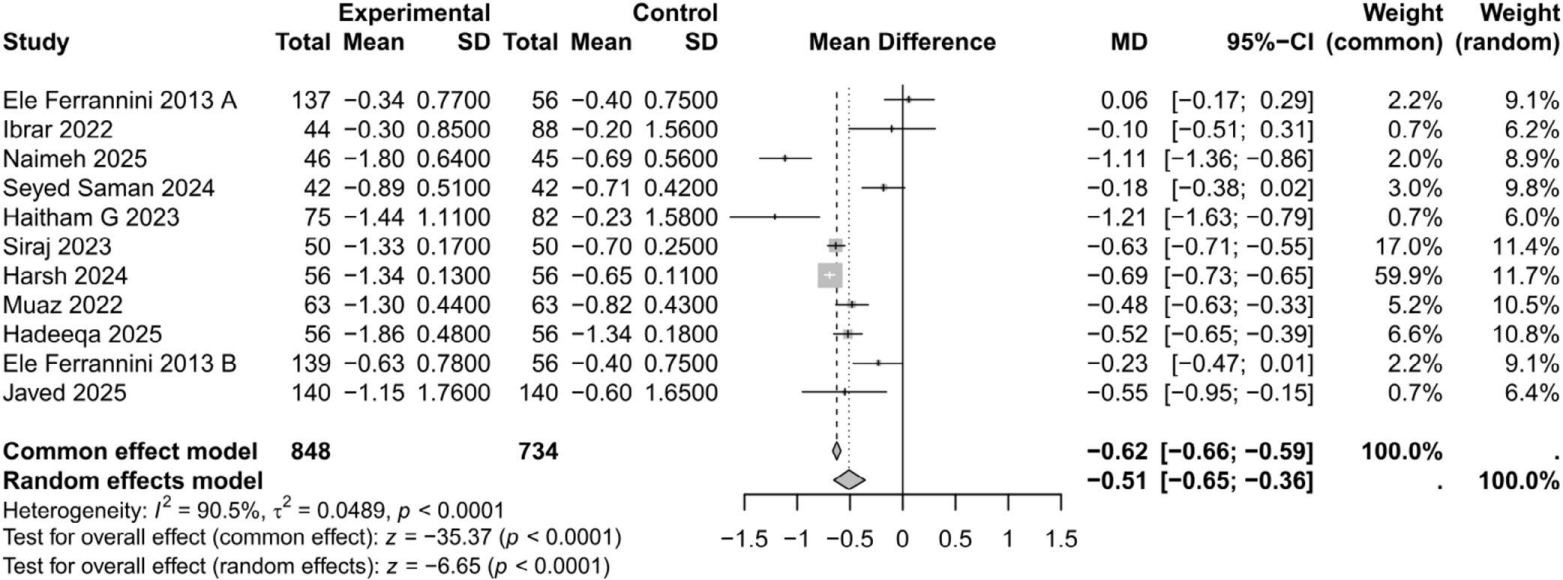
Forest plot for change in HbA1c comparing empagliflozin + metformin versus sitagliptin + metformin.

All 11 studies assessed the change in HbA1c among patients. The meta‐analysis showed a significant reduction in HbA1c (MD −0.51, 95% CI −0.65 to −0.36, *p* < 0.0001) with high heterogeneity (*I* ^2^ = 90.5%) as illustrated in Figure 2. A leave‐one‐out sensitivity analysis, excluding Ele Ferrannini 2013 A, yielded the largest reduction in heterogeneity (*I* ^2^ = 87.5%) (MD = −0.56, 95% CI −0.70 to −0.43, *p* < 0.0001) as shown in Figure S2. Funnel plot analysis and Egger's test indicated no significant asymmetry (*p* = 0.180), suggesting minimal evidence of publication bias as shown in Figure S20.

#### 3.4.2 Change in Body Weight (kg)

**FIGURE 3:**
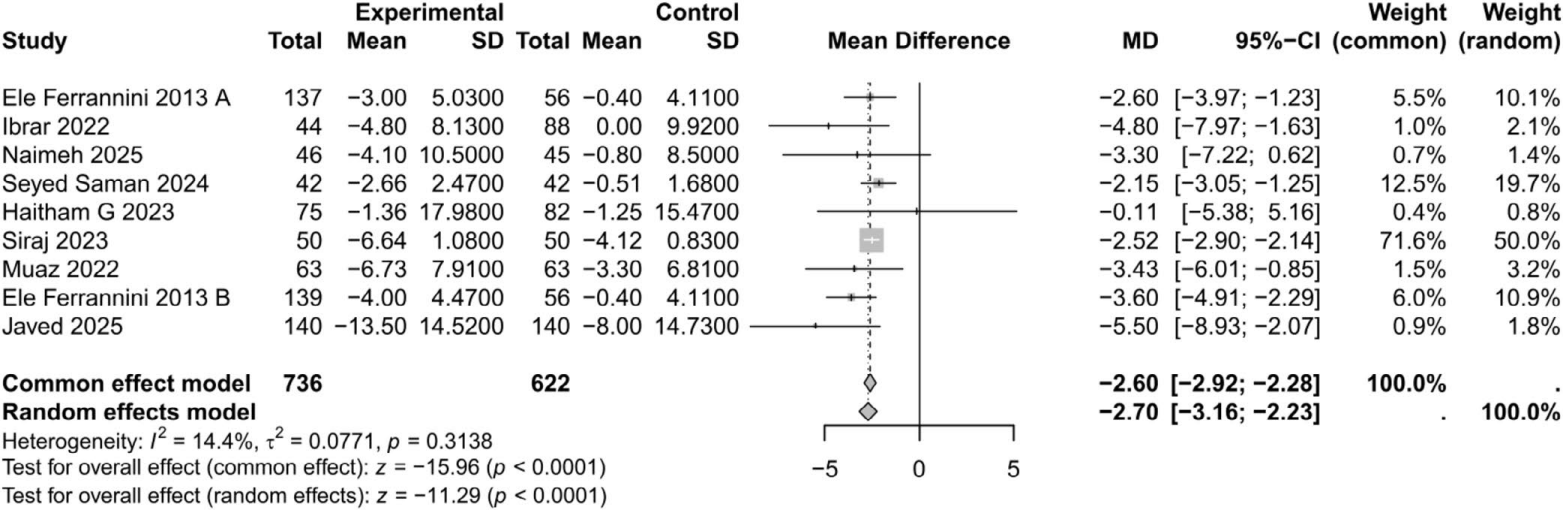
Forest plot for change in body weight comparing empagliflozin + metformin versus sitagliptin + metformin.

Nine of the eleven studies evaluated the change in body weight. The meta‐analysis revealed a significant reduction in body weight (MD −2.70, 95% CI −3.16 to −2.23, *p* < 0.0001) with low heterogeneity (*I* ^2^ = 14.4%) as depicted in Figure 3. Funnel plot evaluation and Egger's test (*p* = 0.180) suggested no significant publication bias as shown in Figure S21.

#### 3.4.3 Change in Total Cholesterol (mg/dL)

**FIGURE 4:**
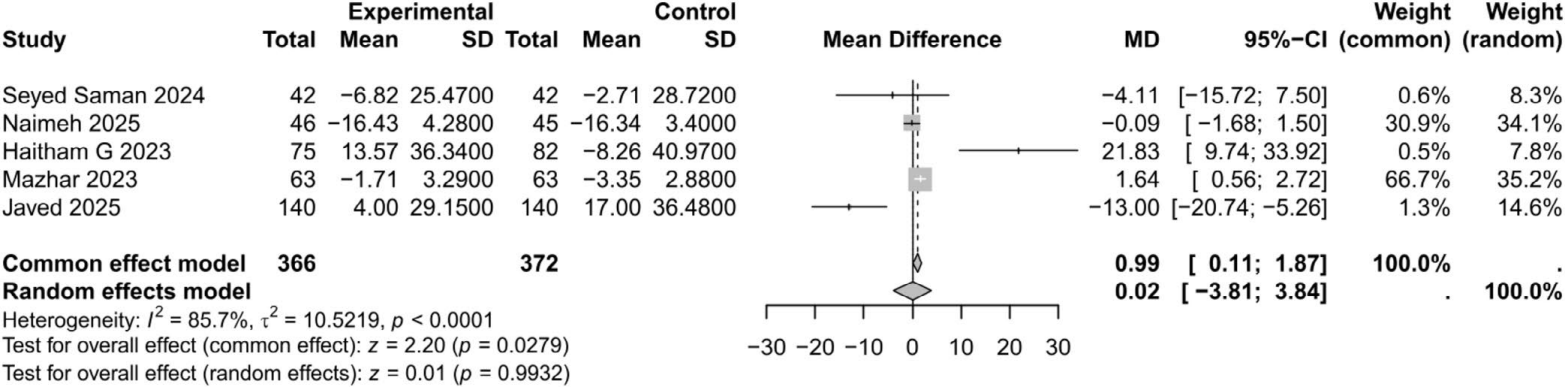
Forest plot for change in total cholesterol comparing empagliflozin + metformin versus sitagliptin + metformin.

Five of the eleven studies assessed changes in total cholesterol levels. The results indicated no significant effect (MD 0.02, 95% CI −3.81 to 3.84, *p* = 0.993) with very high heterogeneity (*I* ^2^ = 85.7%) as shown in Figure 4. A leave‐one‐out sensitivity analysis excluding Javed 2025 reduced heterogeneity the most (*I* ^2^ = 80.2%) (MD 1.17, 95% CI 0.29–2.06, *p* = 0.0096), as shown in Figure S3. The DOI plot revealed an LFK index of −1.33, indicating no major asymmetry and low likelihood of publication bias as shown in Figure S22.

#### 3.4.4 Change in Triglycerides (mg/dL)

**FIGURE 5:**
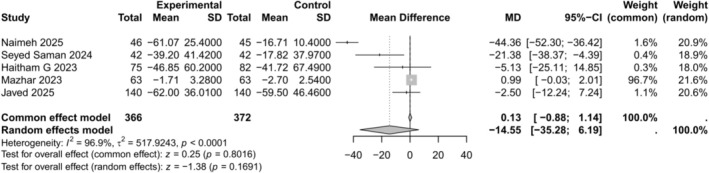
Forest plot for change in triglycerides comparing empagliflozin + metformin versus sitagliptin + metformin.

Five of the eleven studies evaluated the change in triglycerides. The meta‐analysis showed no significant effect (MD −14.55, 95% CI −35.28 to 6.19, *p* = 0.169) with very high heterogeneity (*I* ^2^ = 96.9%) as illustrated in Figure 5. A leave‐one‐out sensitivity analysis excluding Naimeh 2025 reduced heterogeneity the most (*I* ^2^ = 59.7%) (MD 0.86, 95% CI −0.16 to 1.87, *p* = 0.098) as shown in Figure S4. The DOI plot yielded an LFK index of −6.3, suggesting major asymmetry and strong evidence of publication bias as shown in Figure S23.

#### 3.4.5 Change in HDL (mg/dL)

Five of the eleven studies evaluated changes in HDL levels. The meta‐analysis indicated a significant increase (MD 3.61, 95% CI 0.33–6.89, *p* = 0.031) with very high heterogeneity (*I* ^2^ = 99%) as presented in Figure S5. A leave‐one‐out sensitivity analysis excluding Mazhar 2023 reduced heterogeneity the most (*I* ^2^ = 93.4%) (MD 4.12, 95% CI 3.72–4.52, *p* < 0.0001) as shown in Figure S6. The DOI plot produced an LFK index of 5.08, suggesting major asymmetry and strong publication bias as shown in Figure S24.

#### 3.4.6 Change in LDL (mg/dL)

Five of the eleven studies assessed LDL changes. The findings showed a significant reduction (MD −6.43, 95% CI −11.66 to −1.20, *p* = 0.016) with very high heterogeneity (*I* ^2^ = 96.7%) as presented in Figure S7. A leave‐one‐out sensitivity analysis excluding Mazhar 2023 reduced heterogeneity the most (*I* ^2^ = 94.1%) (MD −3.03, 95% CI −3.67 to −2.39, *p* < 0.0001) as shown in Figure S8. The LFK index of −4.52 indicated major asymmetry and strong publication bias as shown in Figure S25.

#### 3.4.7 Change in Systolic Blood Pressure (mmHg)

Five of the eleven studies examined systolic blood pressure changes. The meta‐analysis showed a significant reduction (MD −4.89, 95% CI −7.44 to −2.34, *p* = 0.0002) with substantial heterogeneity (*I* ^2^ = 69.9%) as displayed in Figure S9. A leave‐one‐out sensitivity analysis excluding Javed 2025 reduced heterogeneity the most (*I* ^2^ = 27.9%) (MD −4.27, 95% CI −4.71 to −3.83, *p* < 0.0001) as shown in Figure S10. The LFK index of −2.27 indicated major asymmetry, suggesting strong publication bias as shown in Figure S26.

#### 3.4.8 Change in Diastolic Blood Pressure (mmHg)

Five of the eleven studies evaluated changes in diastolic blood pressure. The meta‐analysis revealed a non‐significant reduction (MD −2.38, 95% CI −6.52 to 1.76, *p* = 0.26) with very high heterogeneity (*I* ^2^ = 94.8%) as shown in Figure S11. A leave‐one‐out sensitivity analysis excluding Naimeh 2025 reduced heterogeneity the most (*I* ^2^ = 77.7%) (MD −0.42, 95% CI −1.55 to 0.72, *p* = 0.47) as shown in Figure S12. The LFK index of 0.9 suggested no significant asymmetry and low publication bias as shown in Figure S27.

#### 3.4.9 Change in Fasting Blood Glucose (mg/dL)

Five of the eleven studies analysed fasting blood glucose. The meta‐analysis indicated a significant reduction (MD −11.73, 95% CI −17.79 to −5.66, *p* = 0.0001) with low‐to‐moderate heterogeneity (*I* ^2^ = 29.8%) as displayed in Figure S13. A leave‐one‐out sensitivity analysis excluding Seyed Saman 2024 reduced heterogeneity the most (*I* ^2^ = 2.1%) (MD −14.32, 95% CI −20.09 to −8.54, *p* < 0.0001) as shown in Figure S14. The LFK index of 0.04 indicated no asymmetry, suggesting minimal publication bias as shown in Figure S28.

### 3.5 Safety Outcomes

#### 3.5.1 UTI

Six of the eleven studies evaluated urinary tract infections. The meta‐analysis revealed no significant difference between groups (RR 0.87, 95% CI 0.52–1.47, *p* = 0.60) with low heterogeneity (*I* ^2^ = 28.8%) as presented in Figure S15. A leave‐one‐out sensitivity analysis excluding Ele Ferrannini 2013 B increased heterogeneity the most (*I* ^2^ = 41.0%) (RR 0.78, 95% CI 0.39–1.55, *p* = 0.47) as shown in Figure S16. The LFK index of −0.83 indicated no significant asymmetry and low publication bias as shown in Figure S29.

#### 3.5.2 Genital Infections

Four of the eleven studies assessed genital infections. The meta‐analysis showed a significantly increased risk in the experimental group (RR 5.93, 95% CI 1.39–25.32, *p* = 0.016) with no heterogeneity (*I* ^2^ = 0%) as shown in Figure S17. The LFK index of 1.68 indicated minor asymmetry and minimal publication bias as shown in Figure S30.

#### 3.5.3 GI Disturbances

Four of the eleven studies evaluated gastrointestinal disturbances. The meta‐analysis indicated a non‐significant reduction (RR 0.59, 95% CI 0.31–1.15, *p* = 0.12) with no heterogeneity (*I* ^2^ = 0%) as presented in Figure S18. The LFK index of −2.65 indicated minor asymmetry and minimal publication bias as shown in Figure S31.

#### 3.5.4 Rash/Allergy

Four of the eleven studies assessed rash incidence. The meta‐analysis demonstrated a significant reduction in rash (RR 0.16, 95% CI 0.04–0.72, *p* = 0.016) with no heterogeneity (*I* ^2^ = 0%) as shown in Figure S19. The LFK index of 2.5 indicated minor asymmetry and minimal publication bias as shown in Figure S32.

### 3.6 Meta‐Regression Analysis

Meta‐regression analysis using a dose of empagliflozin as a moderator suggested a potential dose–response effect, with higher doses showing a trend towards greater body weight reduction (slope = −0.09, 95% CI −0.18 to 0.01) as shown in Figure S33. Although this association did not reach conventional statistical significance (*p* = 0.08), the model explained a large proportion of between‐study variability (*R* ^2^ = 85.0%). These findings indicate that empagliflozin is associated with greater weight loss compared to sitagliptin, the results suggest a potentially greater benefit with higher doses, although further studies are needed to confirm this dose–response relationship.

Moreover, meta‐regression analysis for HbA1c and dose of empagliflozin using dose as a moderator revealed that dose did not significantly influence the treatment effect (coefficient = 0.0075, 95% CI −0.0380 to 0.0531, *p* = 0.75) as shown in Figure S34. The model accounted for 0% of the between‐study variability, and substantial heterogeneity remained unexplained (*I* ^2^ = 96.5%). These findings indicate that empagliflozin reduces HbA1c compared to sitagliptin, and this effect appears consistent across different dose levels as shown in Table S3.

## 4 Discussion

Empagliflozin produced significantly greater glycaemic reductions than sitagliptin across trials. In our meta‐analysis, empagliflozin lowered HbA1c more than sitagliptin. This finding is consistent with prior evidence: meta‐analyses have shown that SGLT2 inhibitors yield meaningfully larger HbA1c reductions than DPP‐4 inhibitors in head‐to‐head comparisons. Min et al. [17] found an adjusted mean difference in HbA1c of −0.24% favouring SGLT2 versus DPP‐4 add‐on therapy to insulin (95% CI −0.43 to −0.05). Similarly, Mishriky et al. [18] reported a small but statistically superior long‐term HbA1c decrease with SGLT2 inhibitors added to metformin (MD = −0.11% at ≥ 52 weeks). In our meta‐analysis of 11 trials, almost all RCTs showed numerically greater HbA1c and fasting glucose reductions with empagliflozin than sitagliptin. Zakaraia et al. [19] found a mean 12‐week HbA1c reduction of −1.13% with empagliflozin (50 mg daily) versus −0.81% with sitagliptin (50 mg, which is lower than the commonly prescribed 100 mg dose for patients with normal renal function). In that trial, fasting glucose also declined more with empagliflozin (*p* = 0.005) [19]. Hiruma et al. [20] similarly reported significantly lower post‐treatment plasma glucose with empagliflozin (≥ 10 mg) than with sitagliptin (*p* < 0.05). These patterns fit the drugs' mechanisms: SGLT2 inhibition promotes renal glucose excretion leading to *slightly* greater glycaemic lowering, whereas DPP‐4 inhibition has more modest effects on insulin/glucagon balance. Overall, the meta‐analysis findings, with empagliflozin yielding a statistically larger HbA1c drop than sitagliptin, are clinically consistent with this pharmacology and prior meta‐analysis [17, 18].

In the meta‐analysis, fasting blood glucose was on average lower with empagliflozin, mirroring HbA1c. The indirect meta‐analysis by Min et al. [17] showed SGLT2 + insulin lowered FPG by 18 mg/dL more than DPP‐4 + insulin. In our trials, most studies reported numerically lower fasting glucose on empagliflozin. Overall, the superiority of empagliflozin on glycaemic endpoints is clinically meaningful and consistent. This aligns with clinical experience that both drugs improve glycaemia, with only slightly greater efficacy for SGLT2 inhibitors [17, 18].

Empagliflozin's weight‐reducing effect was substantially greater than sitagliptin's. SGLT2 inhibitors are well‐known to cause modest weight loss, whereas DPP‐4 inhibitors are generally weight‐neutral [17, 18]. In our meta‐analysis, empagliflozin significantly reduced body weight (mean loss 2–3 kg) compared to sitagliptin. Mishriky et al. reported average weight losses of 2.3–2.5 kg with SGLT2 inhibitors versus DPP‐4, and Min et al. found a −2.38 kg difference in favour of SGLT2 (95% CI −3.18 to −1.58) [17, 18]. These differences are clinically meaningful: even a 2–3 kg additional weight loss can improve metabolic risk factors. Our findings thus echo extensive literature: meta‐analytic evidence consistently finds SGLT2i reduce weight more than DPP‐4i (MD −2 kg).

The meta‐regression explored whether higher empagliflozin dose produced greater weight loss. We found no significant dose–response for body weight, consistent with the idea that SGLT2‐mediated weight loss plateaus at moderate doses. Cai et al. showed that weight reduction with dapagliflozin increased with dose versus placebo, but significant weight loss was already achieved at low dose. The dose‐range analysis by Lim et al. similarly noted weight loss with all SGLT2i and a clear dose–response for some agents [21]. In our analysis, though, empagliflozin 10 versus 25 mg did not produce significantly different weight changes. Only a modest fraction of inter‐study heterogeneity in weight effect was explained by dose variation, suggesting that other factors (baseline BMI, diet, adherence) likely drive differences between trials.

Empagliflozin and sitagliptin had generally neutral but subtly different effects on lipids. SGLT2 inhibitors tend to raise HDL and LDL cholesterol slightly while modestly lowering triglycerides [22]. In contrast, sitagliptin is largely lipid‐neutral or mildly improves HDL and triglycerides [23]. In our meta‐analytic estimates, any differences between empagliflozin and sitagliptin were small. The meta‐analysis by Bechmann et al. [22] confirms class effects: SGLT2 inhibitors increase total and LDL cholesterol and raise HDL while reducing triglycerides. Conversely, Fan et al. [23] found sitagliptin modestly reduced TG (−0.24 mmol/L) and raised HDL (+0.05 mmol/L) with no change in LDL. In practice, these effects are largely offset: empagliflozin's slight LDL rise is balanced by sitagliptin's neutrality, so the net between‐group differences may be negligible. Indeed, our meta‐analysis found no major lipid perturbations. We interpret this as consistent with current evidence: any lipid changes are small and likely not clinically significant [22, 23]. Nonetheless, practitioners should be aware that SGLT2 inhibitors can slightly elevate LDL, so modest increases observed with empagliflozin are expected.

SGLT2 inhibitors are known to reduce systolic BP by 3–5 mmHg on average, whereas DPP‐4 inhibitors have minimal impact [24]. In the meta‐analysis by Zhang et al. [24], SGLT2i reduced SBP by an additional −4.44 mmHg (95% CI −5.33 to −3.55) and DBP by −2.15 mmHg (−2.67 to −1.62) compared to DPP‐4i. Our meta‐analysis results align with these findings: empagliflozin arms showed modest but significantly greater reductions in both SBP and DBP than sitagliptin. The clinical implication is that empagliflozin can confer the additional benefit of BP lowering, an advantage in hypertensive diabetics. Even if the numeric differences appear modest (a few mmHg), they contribute to risk reduction over time. Overall, our finding of superior BP reduction with empagliflozin is consistent with large‐scale trials (EMPA‐REG) and meta‐analysis [19, 24].

The incidence of UTIs was similar between empagliflozin and sitagliptin. SGLT2 inhibitors raise urinary glucose, theoretically predisposing to UTIs. However, our analysis shows no significant increase in UTI risk. Liu et al. [25] reported that SGLT2 inhibitors had a risk ratio of 1.05 (95% CI 0.98–1.12) for UTIs versus control. In our meta‐analysis, the difference in UTIs between empagliflozin and sitagliptin was not statistically significant, mirroring the SGLT2‐class findings. Any observed UTI cases should be considered within background susceptibility. Importantly, this outcome matches broad analyses: FDA warnings had suggested a possible risk, but meta‐analysis has largely shown UTI rates equivalent to comparators [25]. Clinically, we conclude that urinary infection rates are not significantly higher with empagliflozin than sitagliptin in controlled trials.

Empagliflozin was associated with a significantly higher rate of genital mycotic infections. SGLT2 inhibition causes glycosuria, which promotes yeast overgrowth. Robust meta‐analyses document a threefold increased risk of genital infections on SGLT2 inhibitors [25]. Consistent with this, our meta‐analysis found the incidence of genital infections was significantly higher in the empagliflozin arms. According to Zakaraia et al. [19], genital candidiasis occurred in 5.7% of empagliflozin patients versus 0% with sitagliptin. Ahmed et al. similarly found significantly more fungal genital infections with SGLT2i add‐on (*p* < 0.001) [2]. Our meta‐analysis was consistent with this pattern: empagliflozin users had a substantially elevated genital infection rate compared to sitagliptin (often 2%–3% vs. < 1%). These infections were generally mild and treatable (e.g., topical antifungals); none were life‐threatening. Nevertheless, the finding is clinically relevant: patients should be counselled about genital symptoms, and clinicians should monitor and manage these events. Rare but serious adverse events associated with SGLT2 inhibitors, including Fournier's gangrene and lower limb amputations, have been reported in post‐marketing surveillance. These events were not observed in the relatively small trials included in our meta‐analysis and therefore could not be quantitatively assessed.

Renal function is an important factor influencing the efficacy of SGLT2 inhibitors. Because the mechanism of SGLT2 inhibitors depends on promoting urinary glucose excretion through the proximal renal tubule, their glucose‐lowering effect diminishes in patients with moderate to severe renal impairment. Most of the included trials reported baseline renal function and generally excluded individuals with advanced chronic kidney disease (typically eGFR < 45 mL/min/1.73 m^2^). Therefore, the glycaemic benefits observed in this meta‐analysis largely reflect patients with preserved or mildly impaired renal function. Clinicians should be aware that the efficacy of SGLT2 inhibitors may be reduced in patients with significant renal dysfunction.

Gastrointestinal side effects (nausea, diarrhoea, etc.) were uncommon and similar between groups. Neither empagliflozin nor sitagliptin strongly predispose to GI upset at appreciable rates, consistent with earlier studies [19]. Sitagliptin is known to be weight‐neutral and rarely causes GI symptoms; empagliflozin's GI side effects are minimal.

Regarding rash or hypersensitivity, rates were very low in both groups. DPP‐4 inhibitors have been associated with rare cutaneous reactions (including reports of bullous pemphigoid), whereas SGLT2 inhibitors are not known for causing drug rashes. In our trials, allergy/rash events were uncommon (on the order of 1%) and did not differ significantly, consistent with literature [26]. This suggests no clear safety disadvantage for empagliflozin in terms of dermatologic or allergic reactions. Overall, both drugs were well tolerated; the main safety distinction was the observed increase in genital infections with empagliflozin, while other side effects (UTIs, GI, rash) were infrequent and comparable.

### 4.1 Dose–Response Meta‐Regression and Heterogeneity

We conducted meta‐regressions to assess whether higher empagliflozin doses were associated with greater efficacy. For body weight, higher doses were associated with greater weight reduction (slope = −0.09, 95% CI −0.18 to 0.01, *p* = 0.08), and the model explained a large proportion of between‐study variability (*R* ^2^ = 85.0%) (Figure S33). Although this association did not reach conventional statistical significance, the high *R* ^2^ suggests that dose differences may account for much of the observed heterogeneity in weight outcomes. This pattern aligns with pharmacokinetic/pharmacodynamic modelling of SGLT2 inhibitors, which demonstrates diminishing incremental effects at higher doses, particularly for glycosuric and caloric loss mechanisms [27]. Nevertheless, the potential trend observed and limited number of dose‐varying studies mean that these findings remain hypothesis generating; as Pinto et al. [28] reported, dose‐based meta‐regression often explains only part of overall variability in SGLT2 inhibitor outcomes. Further dose‐ranging trials or individual‐patient data meta‐analyses are needed to confirm a true dose–response for weight.

In contrast, meta‐regression for HbA1c showed no influence of empagliflozin dose (coefficient = 0.0075, 95% CI −0.0380 to 0.0531, *p* = 0.75) and the model accounted for 0% of between‐study variability (*R* ^2^ = 0%), with substantial unexplained heterogeneity remaining (*I* ^2^ = 96.5%) (Figure S34). These results indicate that the HbA1c‐lowering effect of empagliflozin appears consistent across the dose range represented in our included trials (primarily 10 mg vs. 25 mg). This is consistent with established SGLT2 class models showing an E‐max of approximately 0.8% for HbA1c reduction [27], indicating that most of the glycaemic benefit is achieved even at standard therapeutic doses and that additional dose escalation yields minimal further HbA1c improvement.

In summary, our meta‐regression suggests a potential trend towards a dose–response for body weight but no dose effect for HbA1c.

### 4.2 Clinical Significance and Interpretation

The meta‐analytic differences between empagliflozin and sitagliptin are generally modest but meaningful in context. Empagliflozin conferred meaningfully greater reductions in HbA1c (on the order of 0.2%–0.3% more on average) and significantly larger weight loss (2–3 kg more). These effects, combined with empiric BP reduction and known cardiovascular benefits of empagliflozin in high‐risk patients, support its preferential use in overweight or hypertensive diabetics. Conversely, sitagliptin, being weight‐neutral and with no added CV benefit, may be preferred when SGLT2 contraindications exist (recurrent genital infections, severe renal impairment). In safety terms, the increased genital infection risk with empagliflozin is consistent with all data and should be balanced against empagliflozin's clinical gains. Importantly, our findings are in line with existing guidelines: recent consensus reports highlight that SGLT2 inhibitors improve glycaemia and weight without increasing hypoglycaemia, and have cardio‐renal advantages, whereas DPP‐4 inhibitors are weight neutral alternatives with a benign side‐effect profile.

### 4.3 Limitations

This meta‐analysis has several limitations that should be considered when interpreting the findings. First, the number of included studies for certain secondary outcomes, including dose–response analyses for HbA1c and body weight, was limited, which may reduce the statistical power and reliability of these estimates. Second, although formal assessments using funnel plots, Egger's test, and DOI plots did not reveal substantial asymmetry for most outcomes, several lipid and blood pressure outcomes demonstrated asymmetry based on LFK indices, suggesting the potential presence of publication bias and underrepresentation of smaller studies with neutral or negative results. Third, differences in study design and treatment regimens may have introduced heterogeneity across the included trials. Notably, the study by Min et al. evaluated SGLT2 and DPP‐4 inhibitors as add‐on therapy to insulin, representing a population with more advanced disease and progressive β‐cell dysfunction, which may influence responsiveness to oral antidiabetic agents compared with patients treated with metformin alone. Finally, variations in sitagliptin dosing across studies and differences in baseline renal function criteria may also influence treatment effects and limit the generalizability of the pooled findings.

## 5 Conclusion

In conclusion, this meta‐analysis suggests that in adults with type 2 diabetes on background metformin, empagliflozin provides meaningfully superior glycaemic control and greater weight loss and blood pressure reduction compared to sitagliptin. These differences align with pharmacology and published literature. Empagliflozin's effect on HbA1c was consistent across the studied doses, achieving a meaningful overall reduction of 0.51% compared to sitagliptin. The proportion of heterogeneity explained by dose was limited; most variability remains attributable to between‐study differences. Clinicians should interpret these findings in context: while both drugs are effective, empagliflozin offers incremental advantages in metabolic parameters and an expected increase in genital infections, whereas sitagliptin provides similar glycaemic control with minimal weight impact.

## Author Contributions

**Saad Ashraf:** conceptualization, investigation, project administration. **Muhammad Burhan:** methodology, data curation, resources. **Sara Sarwar:** validation. **Ahila Ali:** visualization. **Aiza Ahsan:** writing – review and editing. **Shahzad Ashraf:** writing – original draft. **Hammad Javaid:** writing – original draft. **Hamza Irfan:** formal analysis. **Muhammad Hamza Naseer Awan:** resources. **Ajeet Singh:** visualization. **Biruk Demisse Ayalew:** supervision.

## Funding

The authors have nothing to report.

## Ethics Statement

The authors have nothing to report.

## Consent

The authors have nothing to report.

## Conflicts of Interest

The authors declare no conflicts of interest.

## Supporting information


**Figure S1:** Risk of bias assessment for included randomized controlled trials.
**Figure S2:** Leave‐one‐out sensitivity analysis for change in HbA1c.
**Figure S3:** Leave‐one‐out sensitivity analysis for change in total cholesterol.
**Figure S4:** Leave‐one‐out sensitivity analysis for change in triglycerides.
**Figure S5:** Forest plot for change in HDL comparing empagliflozin + metformin versus sitagliptin + metformin.
**Figure S6:** Leave‐one‐out sensitivity analysis for change in HDL.
**Figure S7:** Forest plot for change in LDL comparing empagliflozin + metformin versus sitagliptin + metformin.
**Figure S8:** Leave‐one‐out sensitivity analysis for change in LDL.
**Figure S9:** Forest plot for change in systolic blood pressure comparing empagliflozin + metformin versus sitagliptin + metformin.
**Figure S10:** Leave‐one‐out sensitivity analysis for change in systolic blood pressure.
**Figure S11:** Forest plot for change in diastolic blood pressure comparing empagliflozin + metformin versus sitagliptin + metformin.
**Figure S12:** Leave‐one‐out sensitivity analysis for change in diastolic blood pressure.
**Figure S13:** Forest plot for change in fasting blood glucose comparing empagliflozin + metformin versus sitagliptin + metformin.
**Figure S14:** Leave‐one‐out sensitivity analysis for change in fasting blood glucose.
**Figure S15:** Forest plot for urinary tract infections comparing empagliflozin + metformin versus sitagliptin + metformin.
**Figure S16:** Leave‐one‐out sensitivity analysis for urinary tract infections.
**Figure S17:** Forest plot for genital infections comparing empagliflozin + metformin versus sitagliptin + metformin.
**Figure S18:** Forest plot for gastrointestinal disturbances comparing empagliflozin + metformin versus sitagliptin + metformin.
**Figure S19:** Forest plot for rash/allergy comparing empagliflozin + metformin versus sitagliptin + metformin.
**Figure S20:** Funnel plot for assessing publication bias for change in HbA1c.
**Figure S21:** Funnel plot for assessing publication bias for change in body weight.
**Figure S22:** DOI plot and LFK index for assessing publication bias for change in total cholesterol.
**Figure S23:** DOI plot and LFK index for assessing publication bias for change in triglycerides.
**Figure S24:** DOI plot and LFK index for assessing publication bias for change in HDL.
**Figure S25:** DOI plot and LFK index for assessing publication bias for change in LDL.
**Figure S26:** DOI plot and LFK index for assessing publication bias for change in systolic blood pressure.
**Figure S27:** DOI plot and LFK index for assessing publication bias for change in diastolic blood pressure.
**Figure S28:** DOI plot and LFK index for assessing publication bias for change in fasting blood glucose.
**Figure S29:** DOI plot and LFK index for assessing publication bias for urinary tract infections.
**Figure S30:** DOI plot and LFK index for assessing publication bias for genital infections.
**Figure S31:** DOI plot and LFK index for assessing publication bias for gastrointestinal disturbances.
**Figure S32:** DOI plot and LFK index for assessing publication bias for rash/allergy.
**Figure S33:** Meta‐regression analysis assessing the association between empagliflozin dose and change in body weight.
**Figure S34:** Meta‐regression analysis assessing the association between empagliflozin dose and change in HbA1c.
**Table S1:** Detailed search strategies used for each database.
**Table S2:** NOS table.
**Table S3:** Meta‐regression analysis.

## Data Availability

The datasets generated and/or analysed during this study are available from the corresponding author on reasonable request.
