# Supplementary material for: Glycaemic and Cardiometabolic Outcomes of Empagliflozin Versus Sitagliptin Added to Metformin in T2DM: Insights From a Systematic Review and Meta‐Analysis
Source: Endocrinol Diabetes Metab. 2026 May 12;9(3):e70238. doi: 10.1002/edm2.70238 (PMC13167695; doi:10.1002/edm2.70238)

**Online Supplementary File**

**Supplementary Table 1:** Detailed search strategies used for each database

| **DATABASE** | **SEARCH STRATEGY** | **SEARCH RESULTS** |
| --- | --- | --- |
| **Pubmed** | ("empagliflozin"[Supplementary Concept] OR ("empagliflozin"[Supplementary Concept] OR "empagliflozin"[All Fields]) OR ("empagliflozin"[Supplementary Concept] OR "empagliflozin"[All Fields] OR "bi 10773"[All Fields]) OR ("empagliflozin"[Supplementary Concept] OR "empagliflozin"[All Fields] OR "jardiance"[All Fields])) AND ("sitagliptin phosphate"[MeSH Terms] OR ("sitagliptin phosphate"[Supplementary Concept] OR "sitagliptin phosphate"[All Fields] OR "sitagliptin"[All Fields] OR "sitagliptin phosphate"[MeSH Terms] OR ("sitagliptin"[All Fields] AND "phosphate"[All Fields]) OR "sitagliptine"[All Fields] OR "sitagliptin s"[All Fields]) OR ("sitagliptin phosphate"[Supplementary Concept] OR "sitagliptin phosphate"[All Fields] OR "sitagliptin phosphate"[MeSH Terms] OR ("sitagliptin"[All Fields] AND "phosphate"[All Fields])) OR ("sitagliptin phosphate"[Supplementary Concept] OR "sitagliptin phosphate"[All Fields] OR "januvia"[All Fields] OR "sitagliptin phosphate"[MeSH Terms] OR ("sitagliptin"[All Fields] AND "phosphate"[All Fields]) OR "sitagliptin"[All Fields] OR "sitagliptine"[All Fields] OR "sitagliptin s"[All Fields]) OR ("sitagliptin phosphate"[Supplementary Concept] OR "sitagliptin phosphate"[All Fields] OR "mk 0431"[All Fields] OR "sitagliptin phosphate"[MeSH Terms] OR ("sitagliptin"[All Fields] AND "phosphate"[All Fields]))) AND ("metformin"[MeSH Terms] OR ("metformin"[Supplementary Concept] OR "metformin"[All Fields] OR "metformin"[MeSH Terms] OR "metformine"[All Fields] OR "metformin s"[All Fields] OR "metformins"[All Fields]) OR ("metformin"[Supplementary Concept] OR "metformin"[All Fields] OR "metformin hydrochloride"[All Fields] OR "metformin"[MeSH Terms] OR ("metformin"[All Fields] AND "hydrochloride"[All Fields])) OR ("metformin"[Supplementary Concept] OR "metformin"[All Fields] OR "glucophage"[All Fields] OR "metformin"[MeSH Terms] OR "metformine"[All Fields] OR "metformin s"[All Fields] OR "metformins"[All Fields])) AND ("diabetes mellitus, type 2"[MeSH Terms] OR "type 2 diabetes"[All Fields] OR "T2DM"[All Fields] OR ("diabetes mellitus, type 2"[MeSH Terms] OR "type 2 diabetes mellitus"[All Fields] OR ("noninsulin"[All Fields] AND "dependent"[All Fields] AND "diabetes"[All Fields]) OR "noninsulin dependent diabetes"[All Fields])) | 66 |
| **Embase** | ('diabetes mellitus type 2'/exp OR 'type 2 diabetes' OR 'type ii diabetes' OR t2dm OR 'non insulin dependent diabetes mellitus' OR niddm) AND ('empagliflozin'/exp OR empagliflozin OR 'bi 10773' OR bi10773 OR jardiance) AND ('sitagliptin'/exp OR sitagliptin OR 'sitagliptin phosphate' OR 'sitagliptin phosphate monohydrate' OR januvia OR 'mk 0431' OR mk0431) AND ('metformin'/exp OR metformin OR 'metformin hydrochloride' OR 'metformin hcl' OR glucophage) AND ('randomized controlled trial'/exp OR randomized OR placebo) | 254 |
| **Cochrane** | ( empagliflozin OR "BI 10773" OR BI10773 OR Jardiance ) AND ( sitagliptin OR "sitagliptin phosphate" OR "sitagliptin phosphate monohydrate" OR Januvia OR "MK-0431" OR MK0431 ) AND ( metformin OR "metformin hydrochloride" OR "metformin HCl" OR Glucophage ) AND ( "type 2 diabetes" OR "type II diabetes" OR "non–insulin dependent diabetes" OR T2DM ) | 34 |
| **Scopus** | TITLE-ABS-KEY ( empagliflozin OR "BI 10773" OR BI10773 OR Jardiance ) AND TITLE-ABS-KEY ( sitagliptin OR "sitagliptin phosphate" OR "sitagliptin phosphate monohydrate" OR Januvia OR "MK-0431" ) AND TITLE-ABS-KEY ( metformin OR "metformin hydrochloride" OR "metformin HCl" OR Glucophage ) AND TITLE-ABS-KEY ( "type 2 diabetes" OR "type II diabetes" OR T2DM OR "non insulin dependent diabetes" ) AND TITLE-ABS-KEY ( random* ) | 734 |
| **Clinical trial gov** | (empagliflozin OR BI 10773 OR Jardiance) AND (sitagliptin OR "sitagliptin phosphate" OR Januvia OR "MK-0431") AND (metformin OR "metformin hydrochloride" OR Glucophage) AND ("type 2 diabetes" OR T2DM) | 18 |

**Supplementary Table 2: NOS table**

| Study | Representativeness of the exposed cohort（1） | Selection of the non-exposed cohort (1） | Ascertainment of exposure (1) | Demonstration that outcome of interest was not present at start of study（1） | Comparability of cohorts based on the design or analysis (2) | Assessment of outcome （1） | Was follow up long enough for outcomes to occur（1） | Adequacy of follow up of cohorts（1） |  |
| --- | --- | --- | --- | --- | --- | --- | --- | --- | --- |
| Ibrar et al 2022 | ☆ | ☆ | ☆ | ☆ | ☆ | ☆ | ☆ | ☆ |  |
| Talebi et al 2024 | ☆ | ☆ | ☆ | ☆ | ☆ | ☆ | ☆ | ☆ |  |
|  |  |  |  |  |  |  |  |  |  |

**Supplementary Table 3: Meta-regression analysis**

| **Model** | **k** | **I²** | **R²** | **QM (p)** | **Dose (p)** |
| --- | --- | --- | --- | --- | --- |
| **Dose vs Bodyweight** | 9 | 0.0% | 85.0% | 3.08 (p=0.080) | 0.080 · |
| **HbA1c vs Dose** | 11 | 96.5% | 0.0% | 0.11 (p=0.746) | 0.746 |

**Supplementary Figure 1:** Risk of bias assessment for included randomized controlled trials.


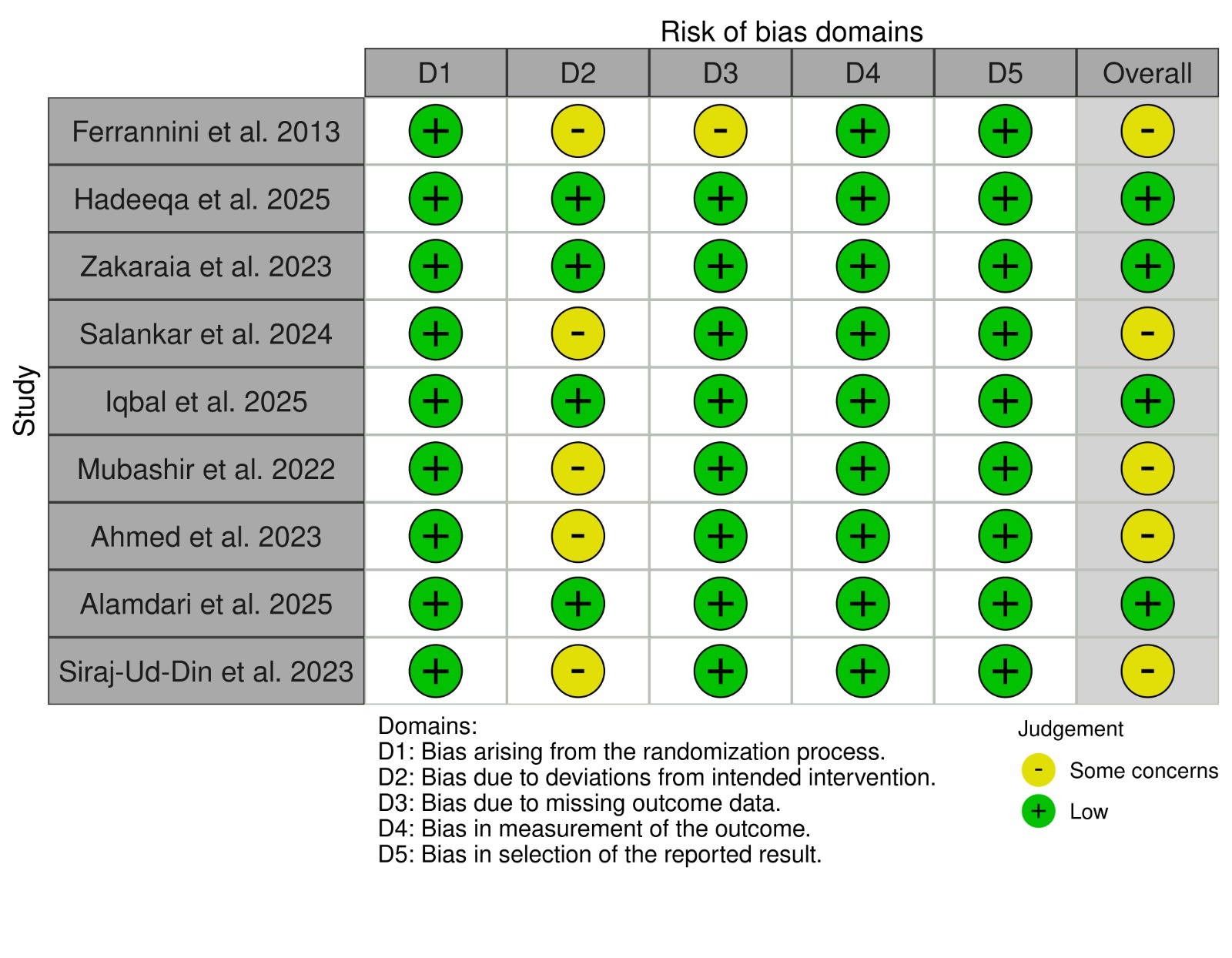


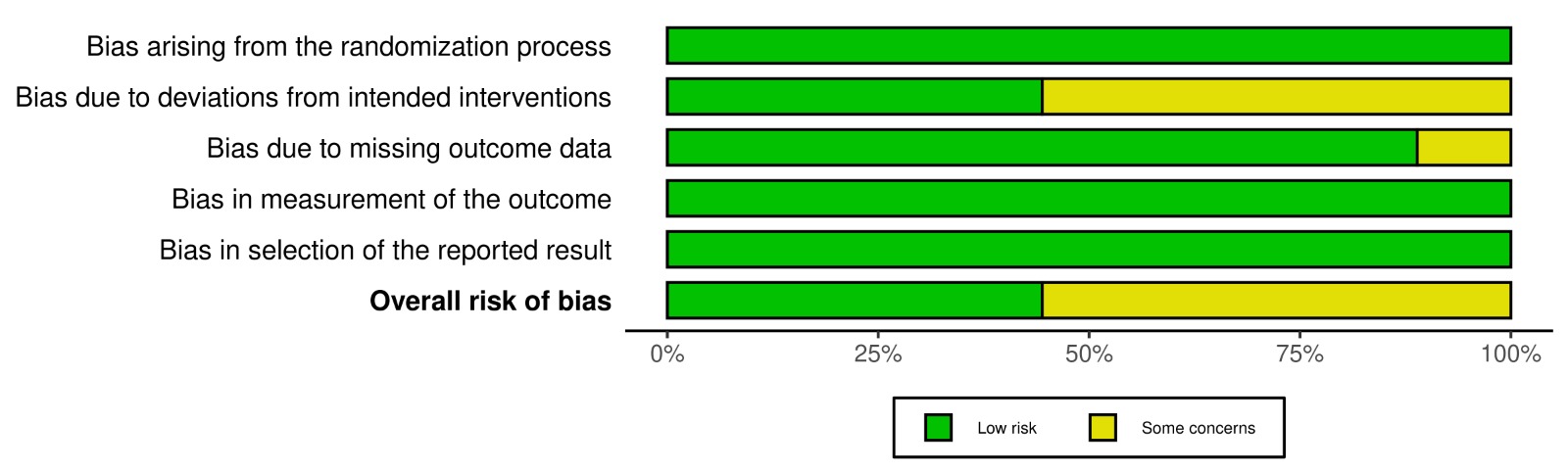


**Supplementary Figure 2:** Leave-one-out sensitivity analysis for change in HbA1c.


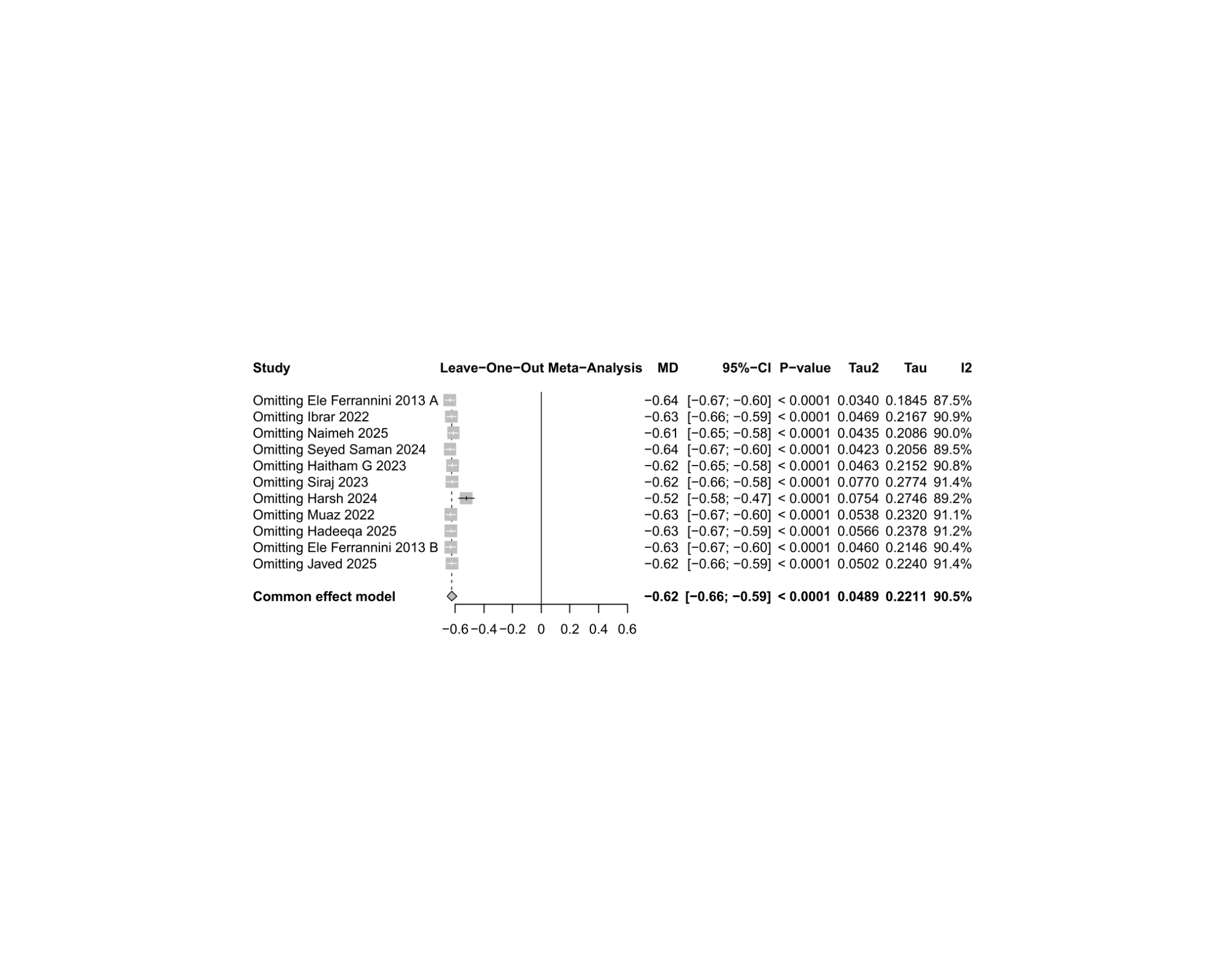


**Supplementary Figure 3:** Leave-one-out sensitivity analysis for change in total cholesterol.


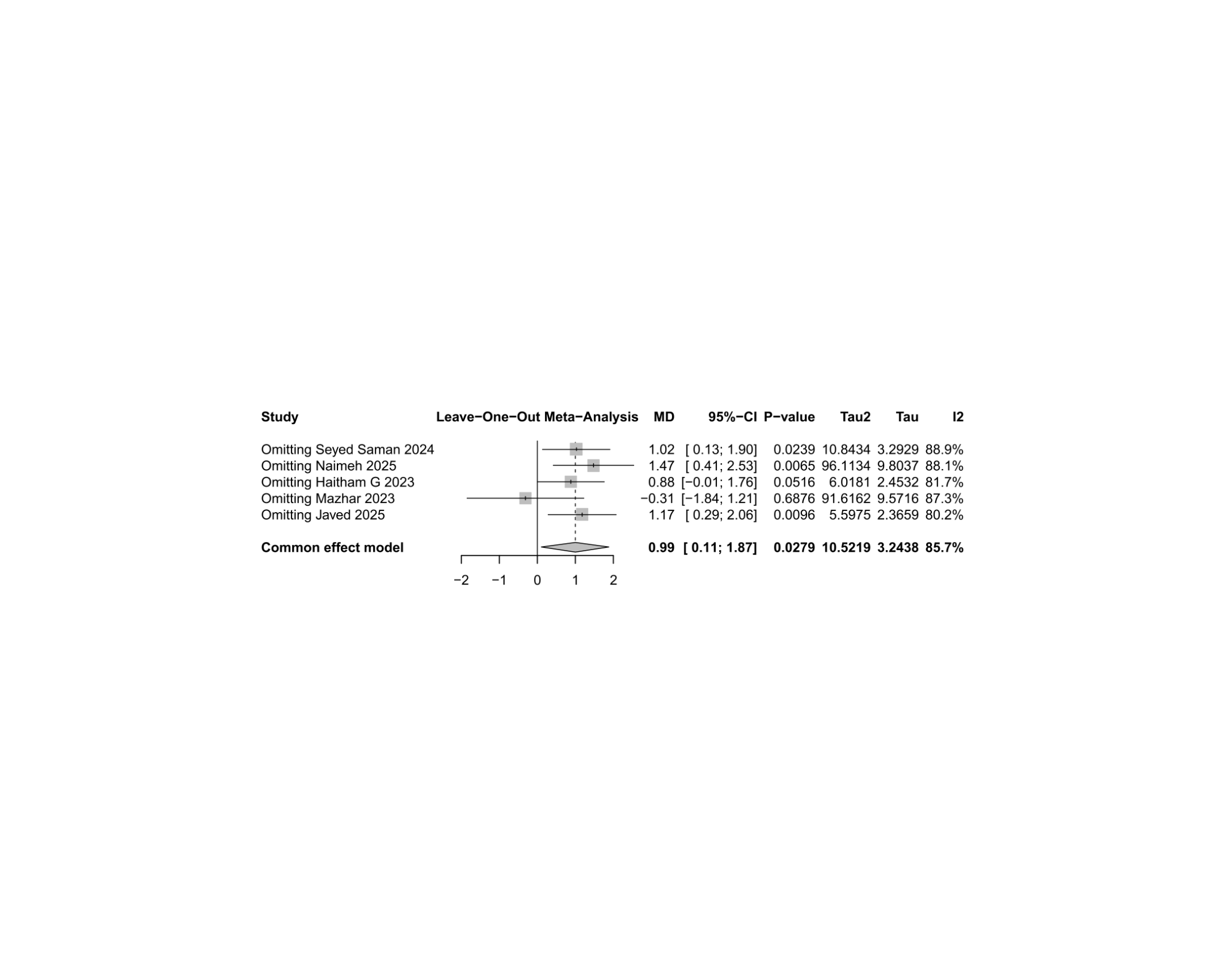


**Supplementary Figure 4:** Leave-one-out sensitivity analysis for change in triglycerides.


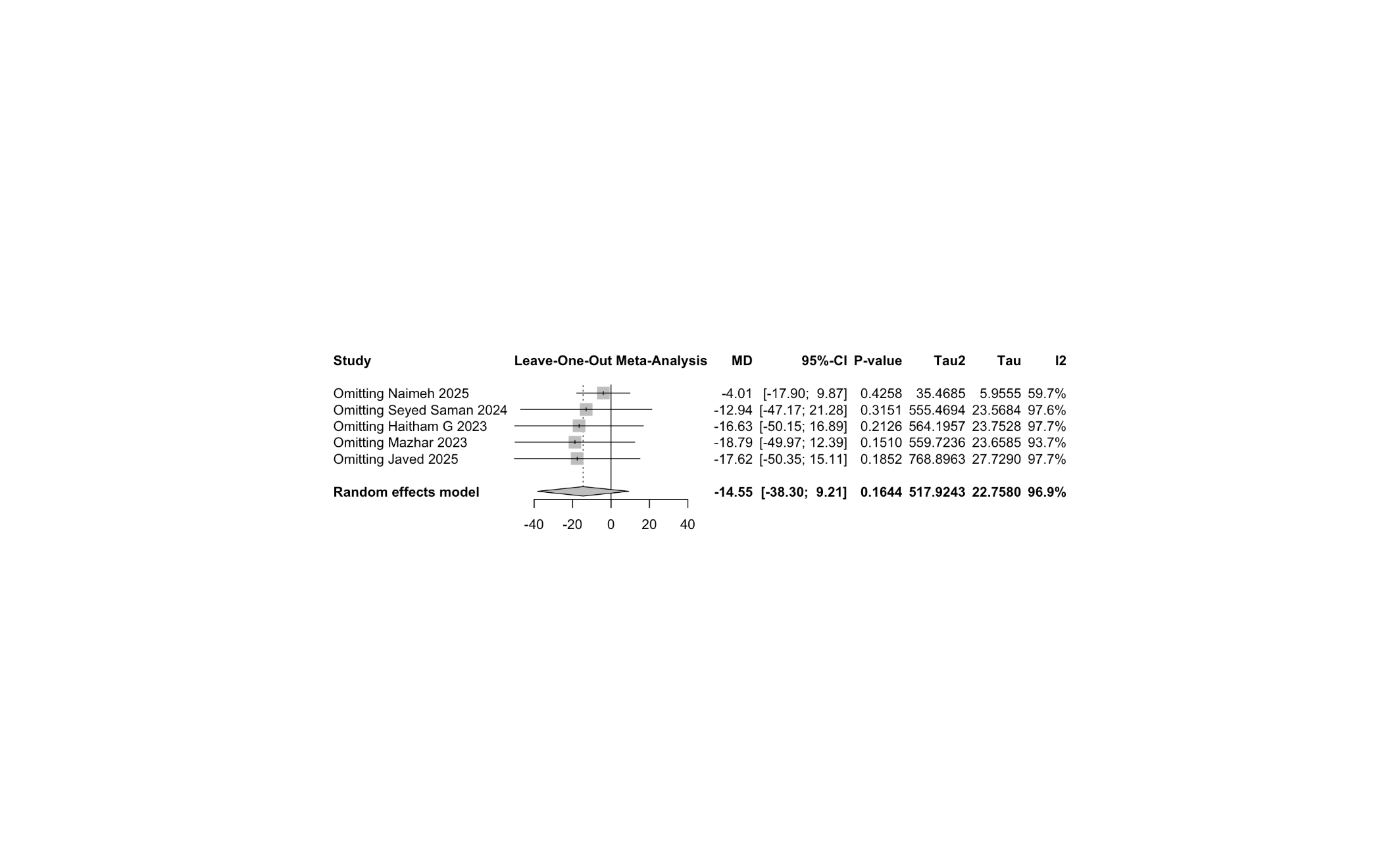


**Supplementary Figure 5:** Forest plot for change in HDL comparing empagliflozin + metformin vs sitagliptin + metformin.


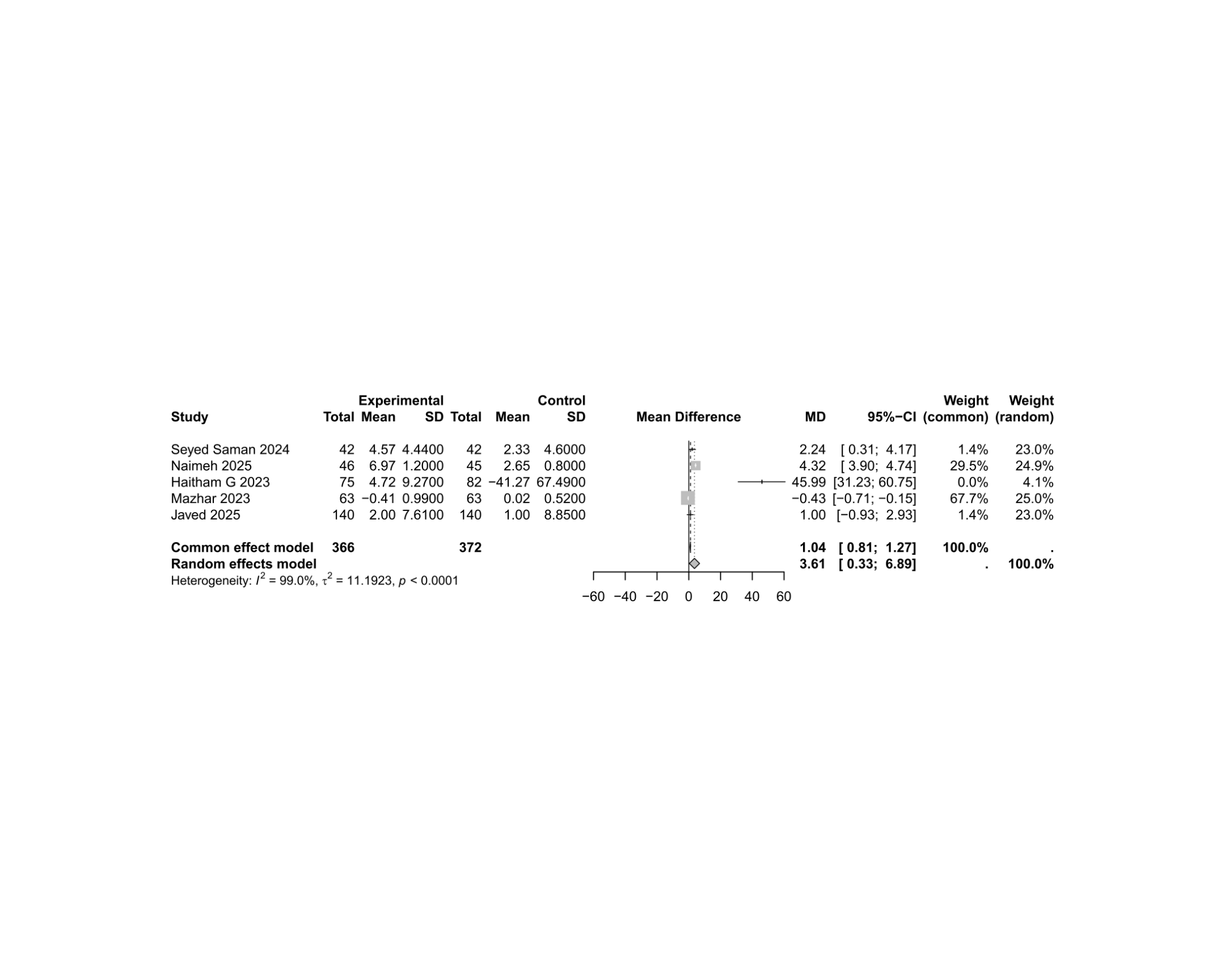


**Supplementary Figure 6:** Leave-one-out sensitivity analysis for change in HDL.


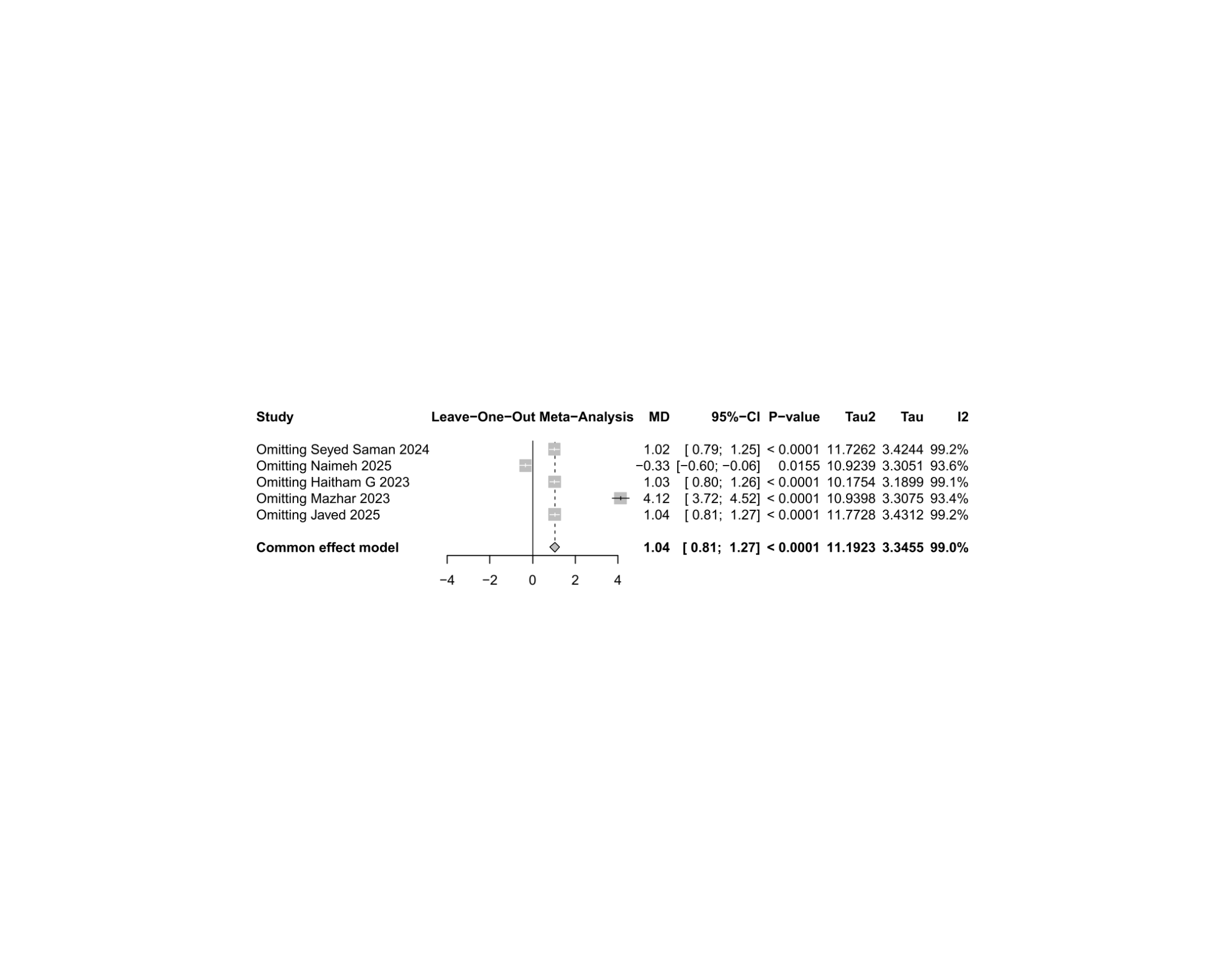


**Supplementary Figure 7:** Forest plot for change in LDL comparing empagliflozin + metformin vs sitagliptin + metformin.


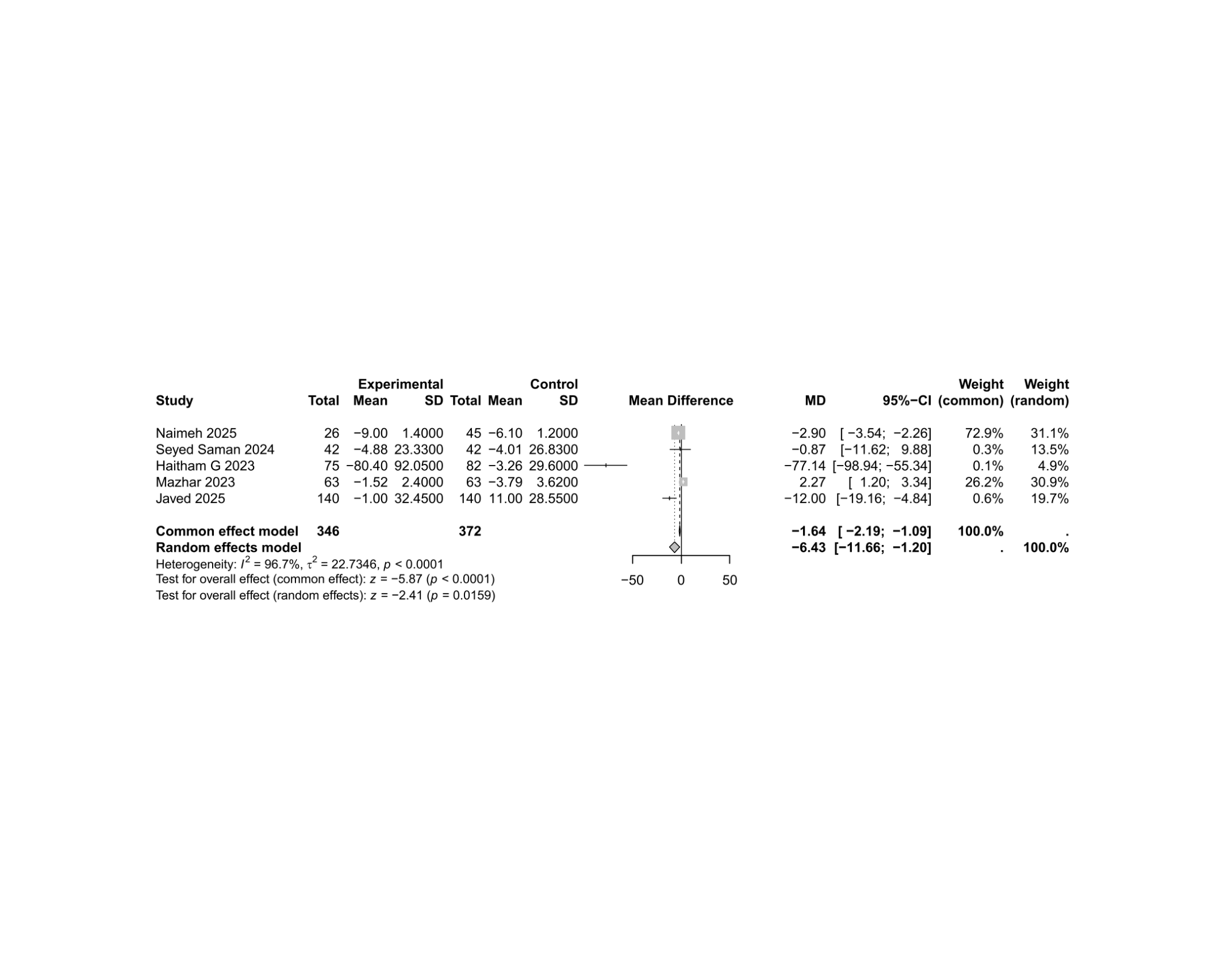


**Supplementary Figure 8:** Leave-one-out sensitivity analysis for change in LDL.


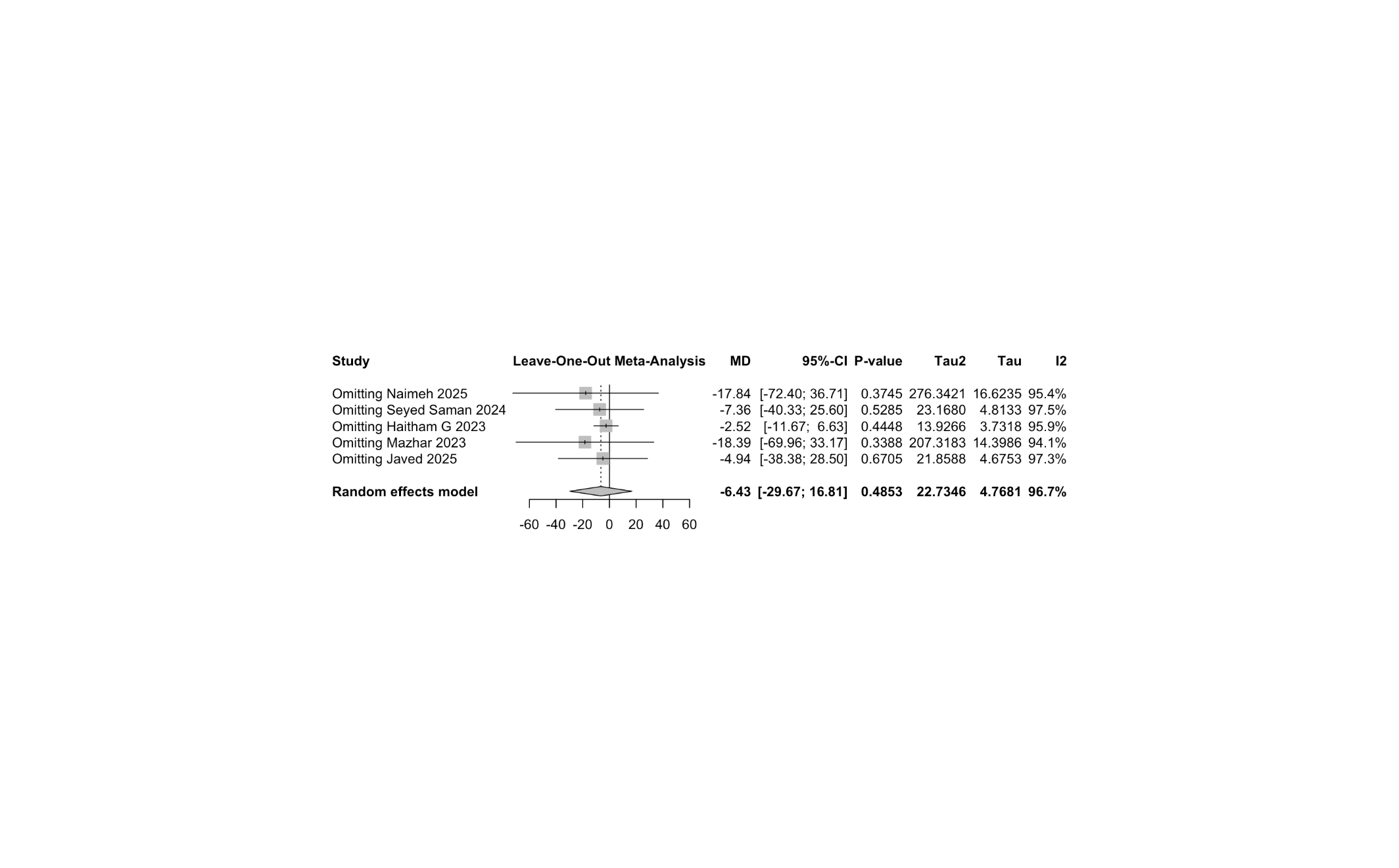


**Supplementary Figure 9:** Forest plot for change in systolic blood pressure comparing empagliflozin + metformin vs sitagliptin + metformin.


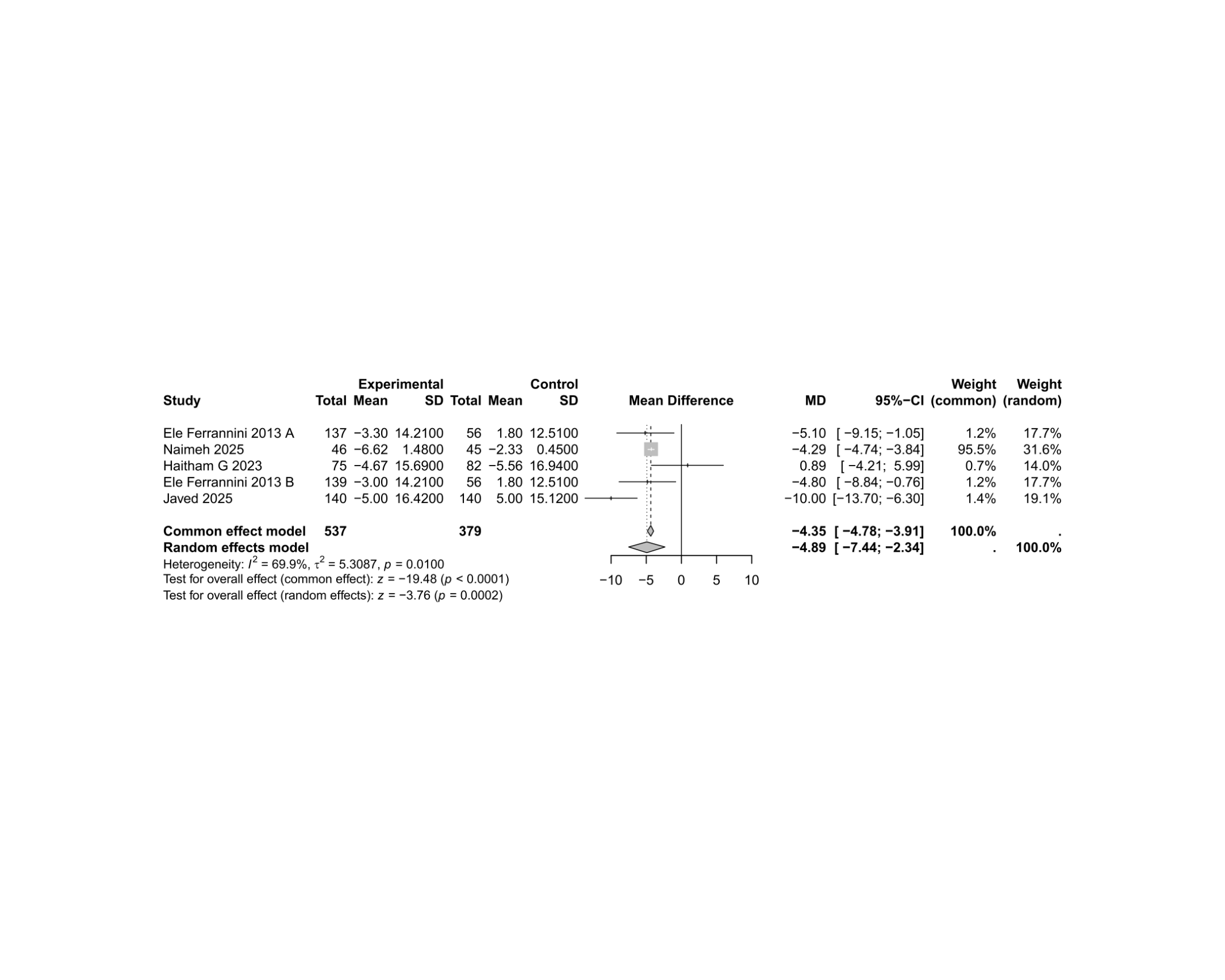


**Supplementary Figure 10:** Leave-one-out sensitivity analysis for change in systolic blood pressure.


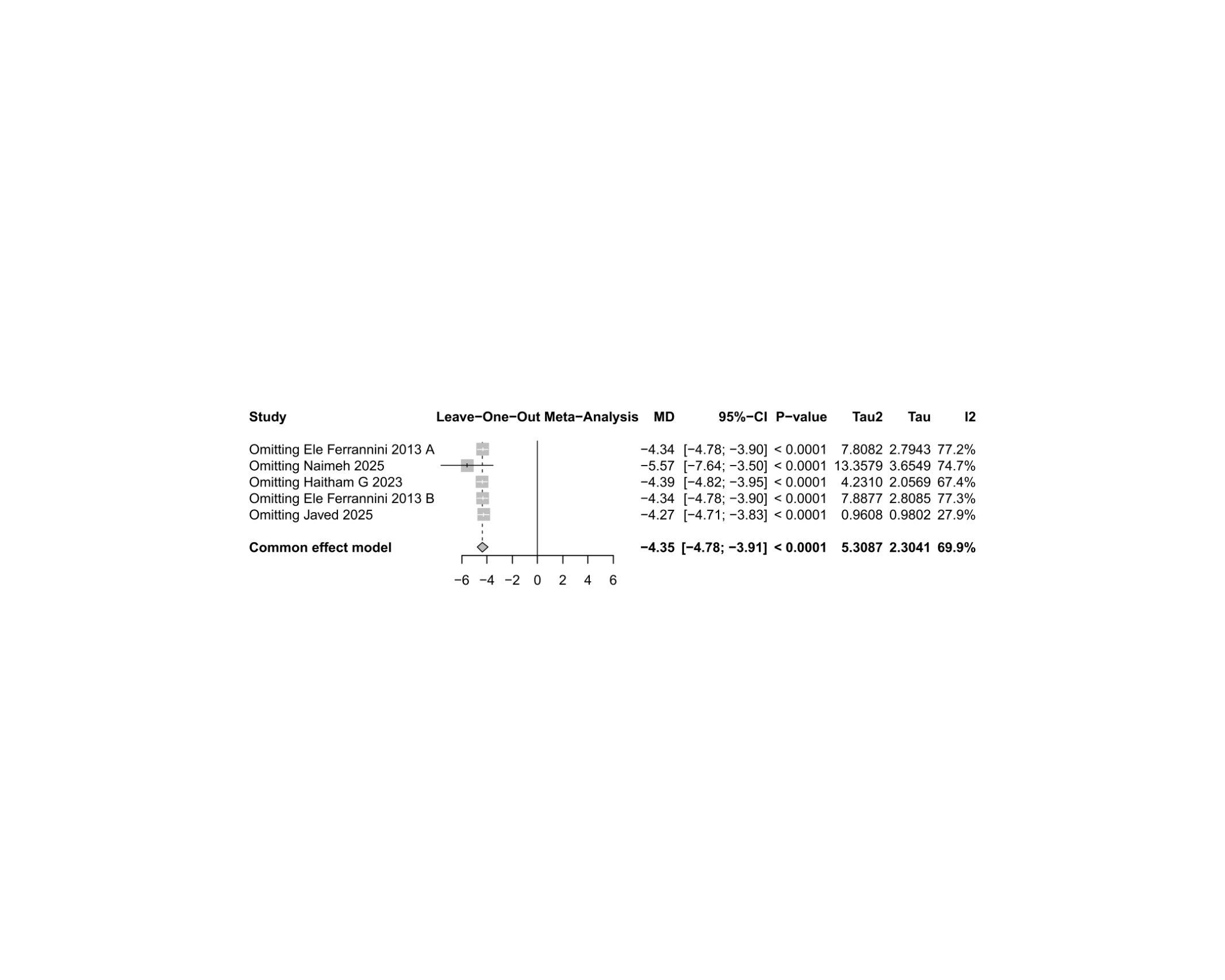


**Supplementary Figure 11:** Forest plot for change in diastolic blood pressure comparing empagliflozin + metformin vs sitagliptin + metformin.


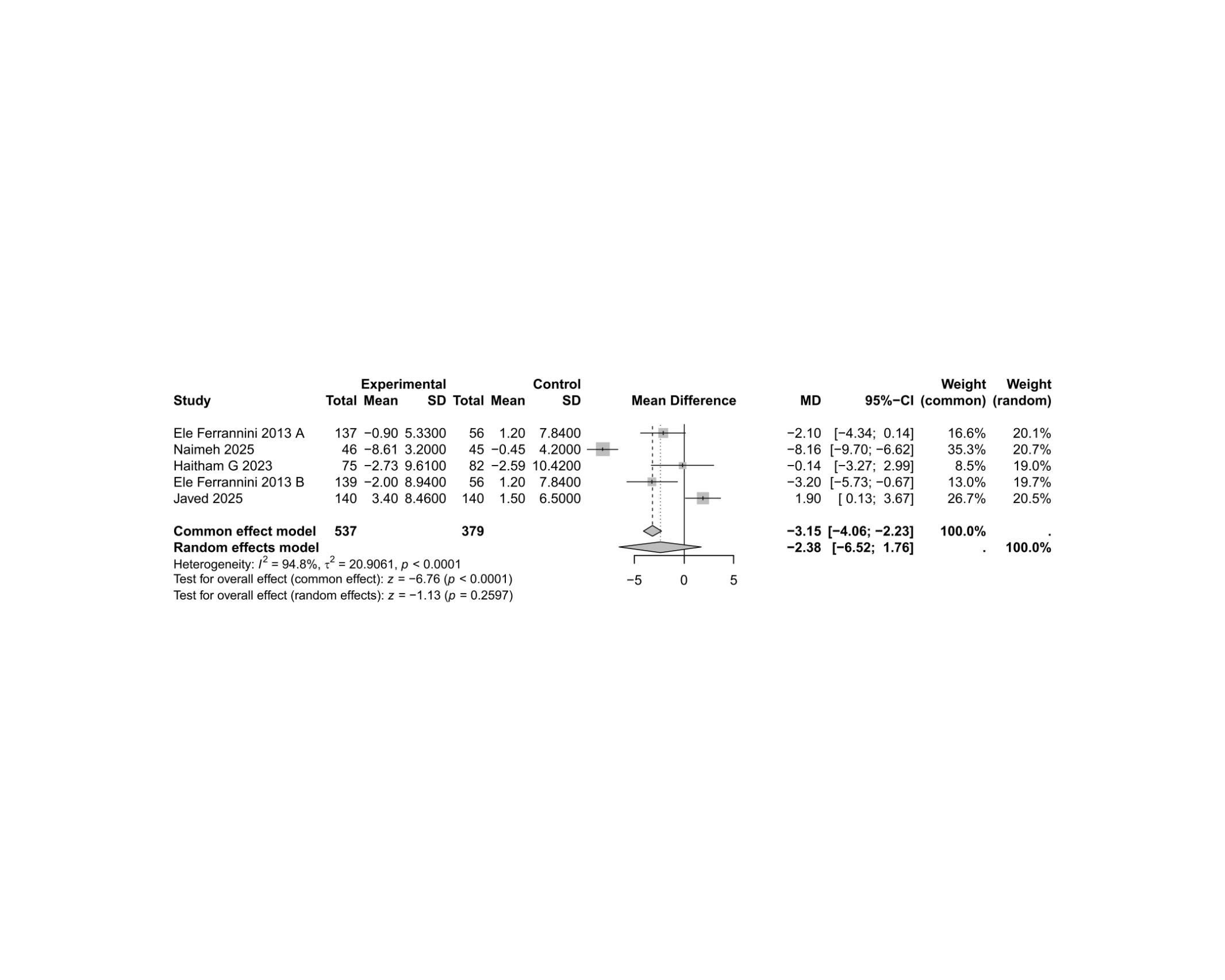


**Supplementary Figure 12:** Leave-one-out sensitivity analysis for change in diastolic blood pressure.


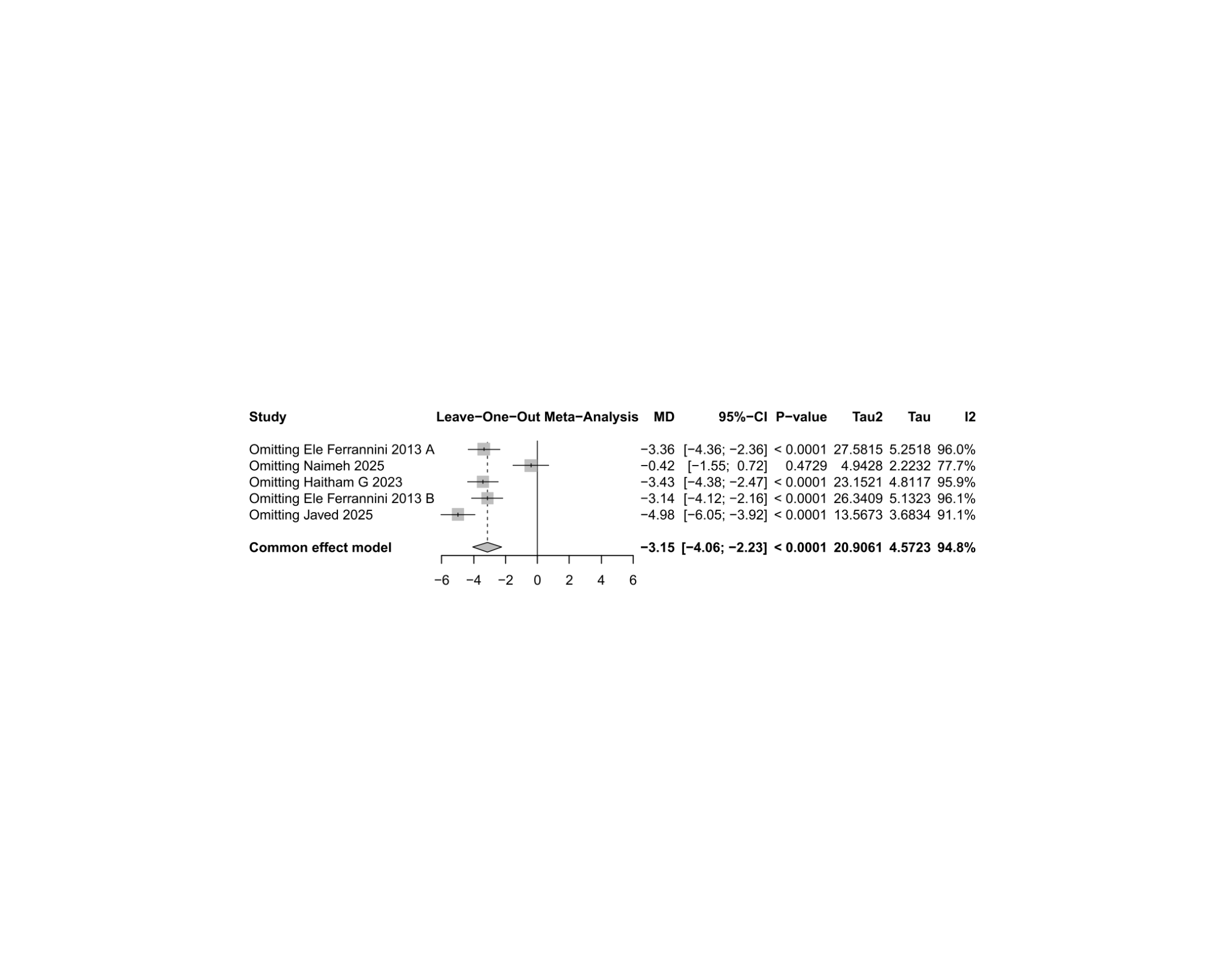


**Supplementary Figure 13:** Forest plot for change in fasting blood glucose comparing empagliflozin + metformin vs sitagliptin + metformin.


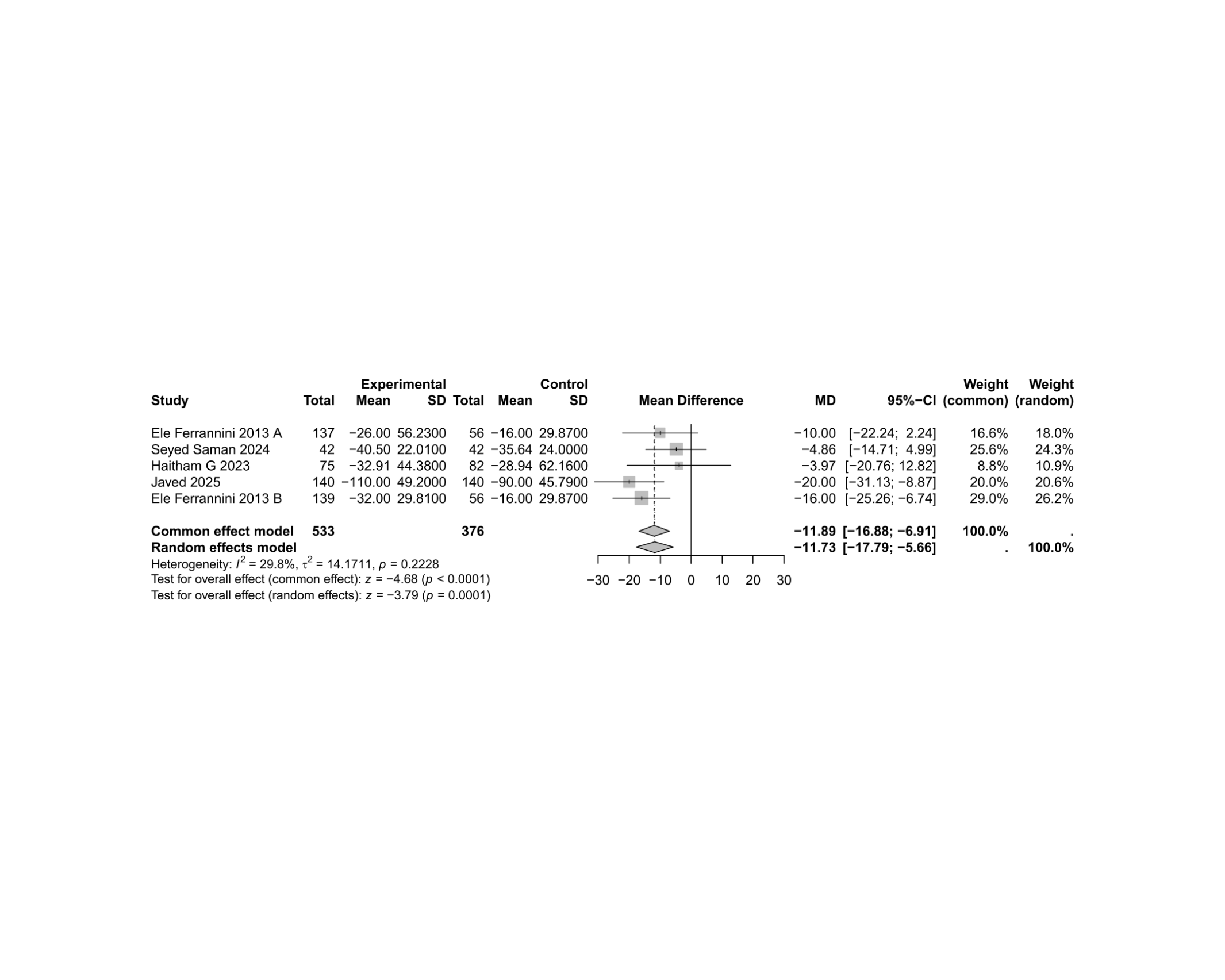


**Supplementary Figure 14:** Leave-one-out sensitivity analysis for change in fasting blood glucose.
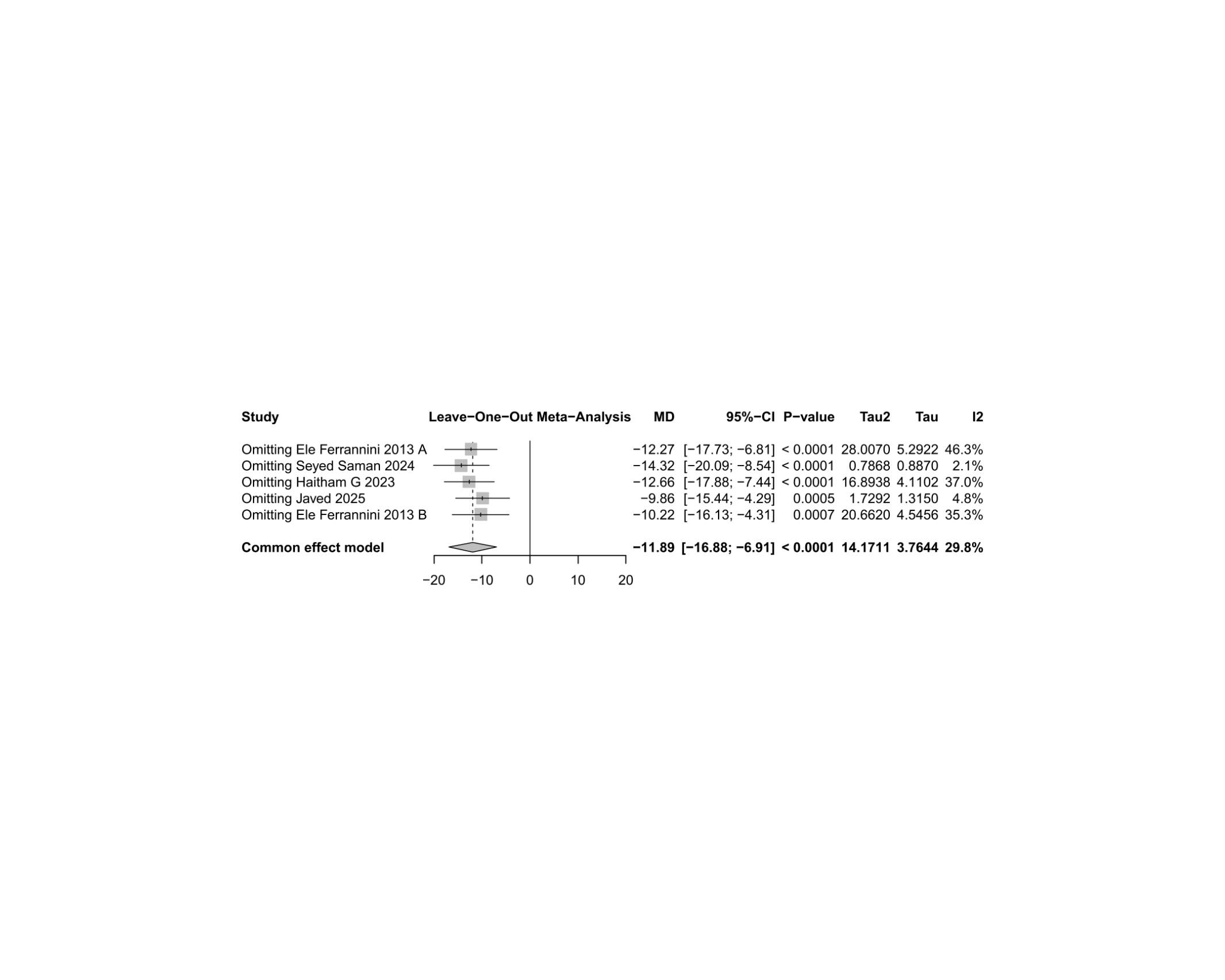


**Supplementary Figure 15:** Forest plot for urinary tract infections comparing empagliflozin + metformin vs sitagliptin + metformin.
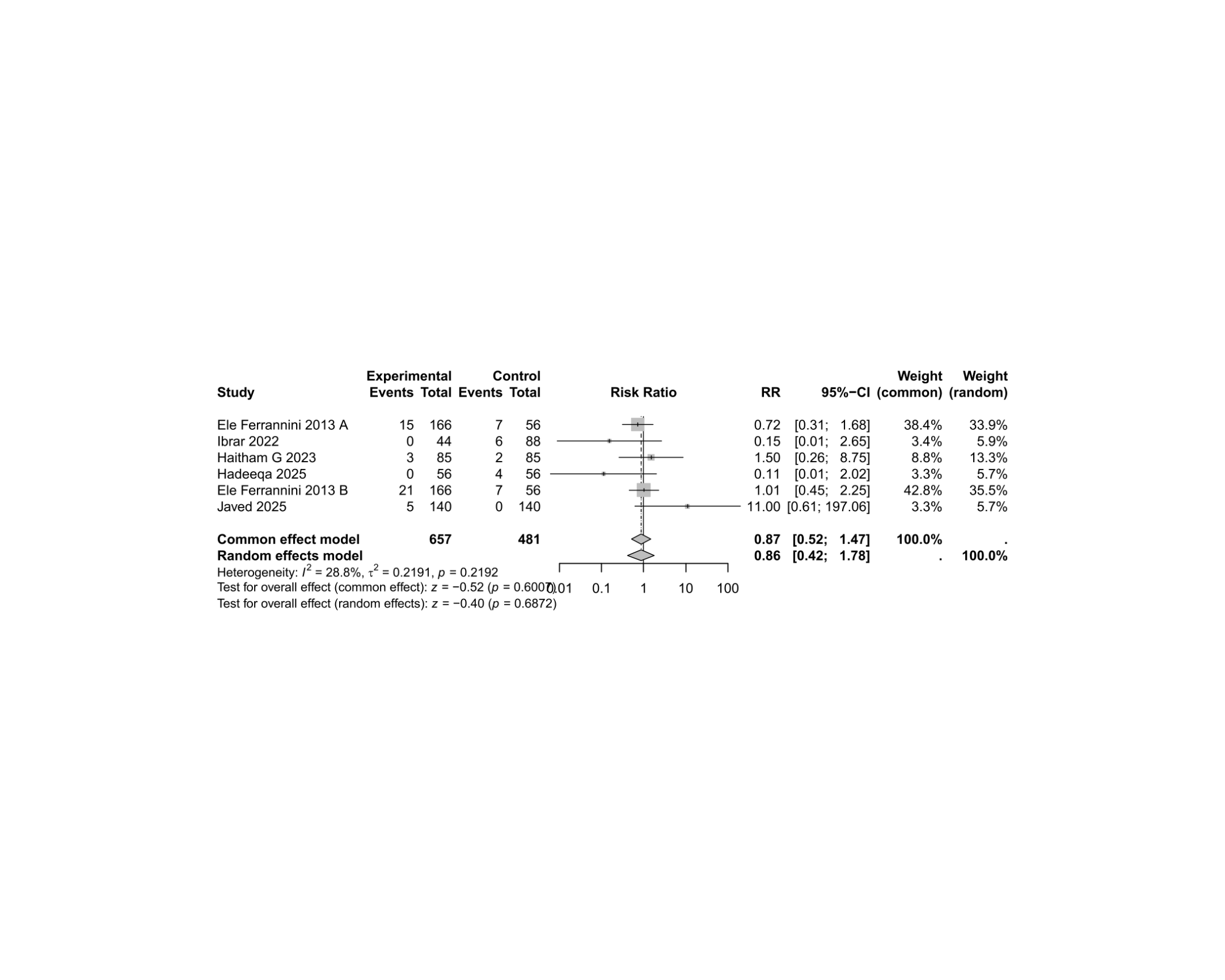


**Supplementary Figure 16:** Leave-one-out sensitivity analysis for urinary tract infections.
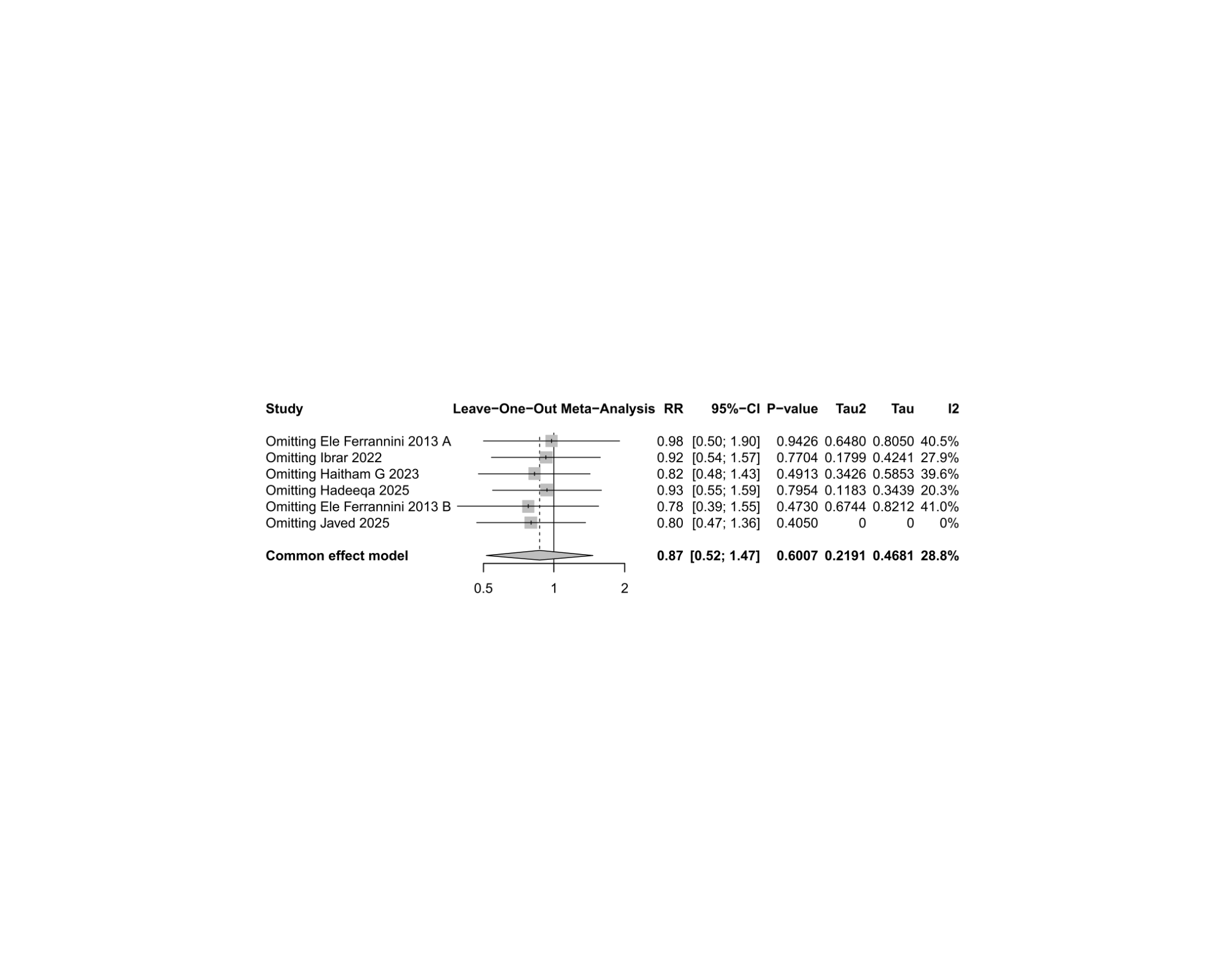


**Supplementary Figure 17:** Forest plot for genital infections comparing empagliflozin + metformin vs sitagliptin + metformin.
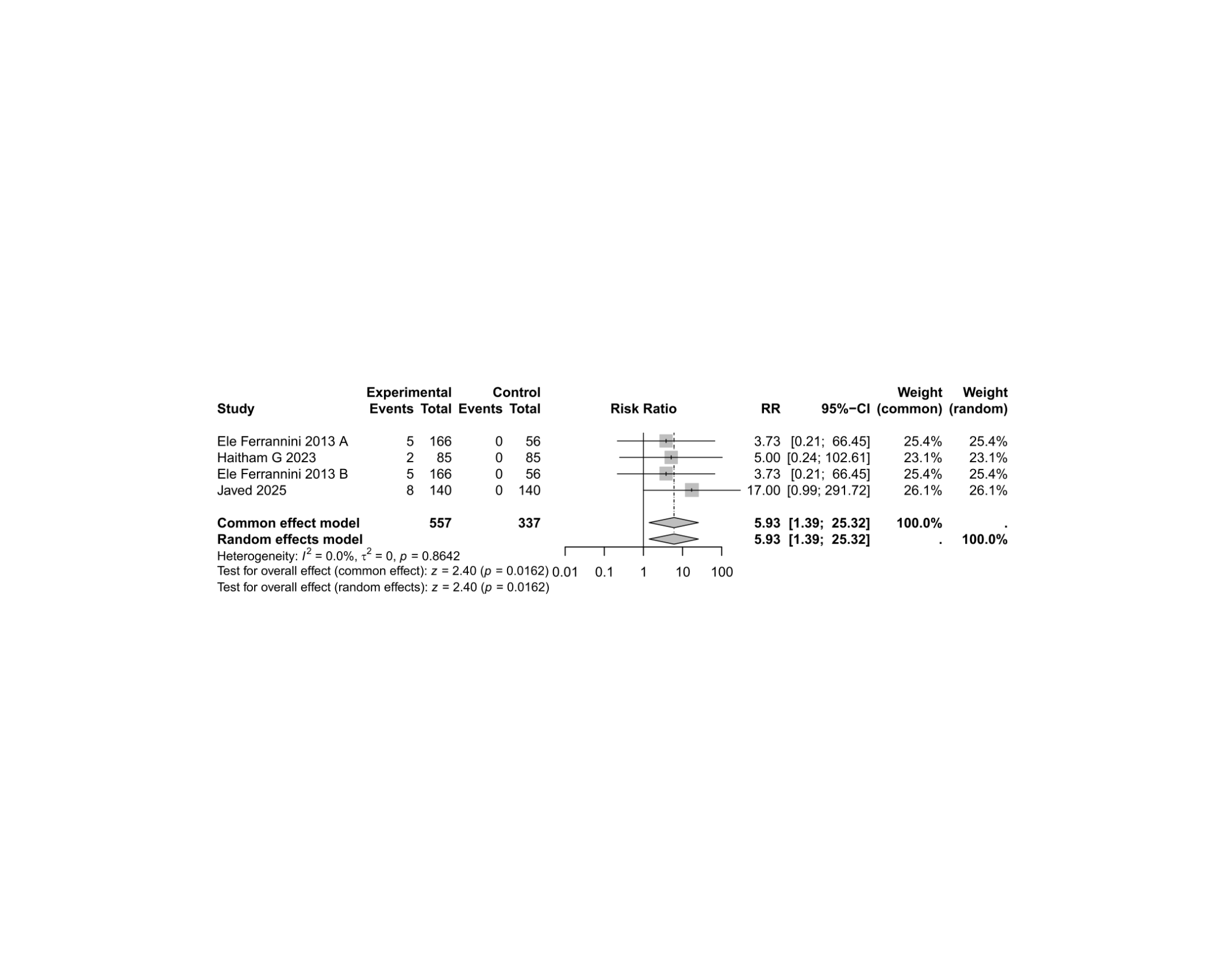


**Supplementary Figure 18:** Forest plot for gastrointestinal disturbances comparing empagliflozin + metformin vs sitagliptin + metformin.
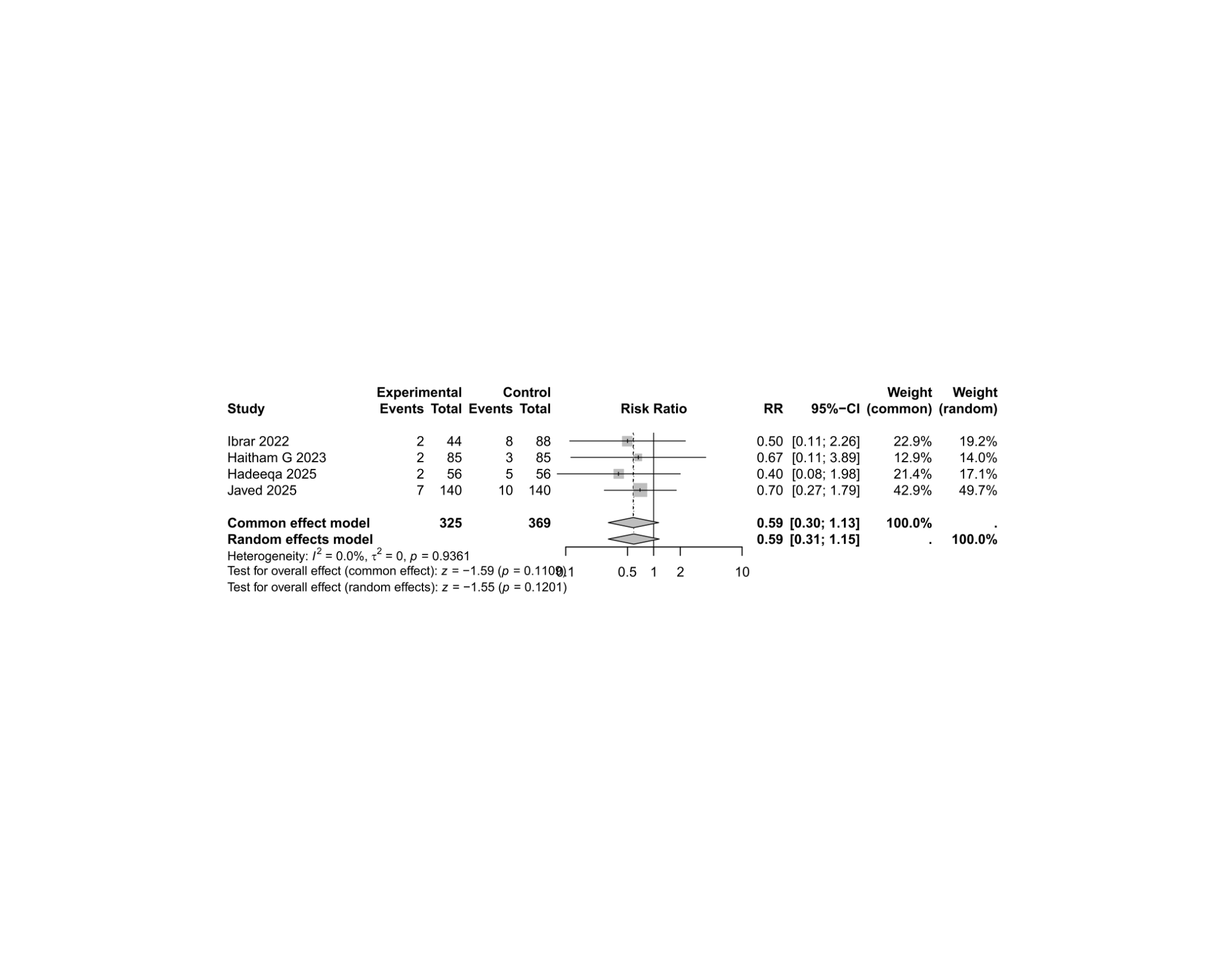


**Supplementary Figure 19:** Forest plot for rash/allergy comparing empagliflozin + metformin vs sitagliptin + metformin.


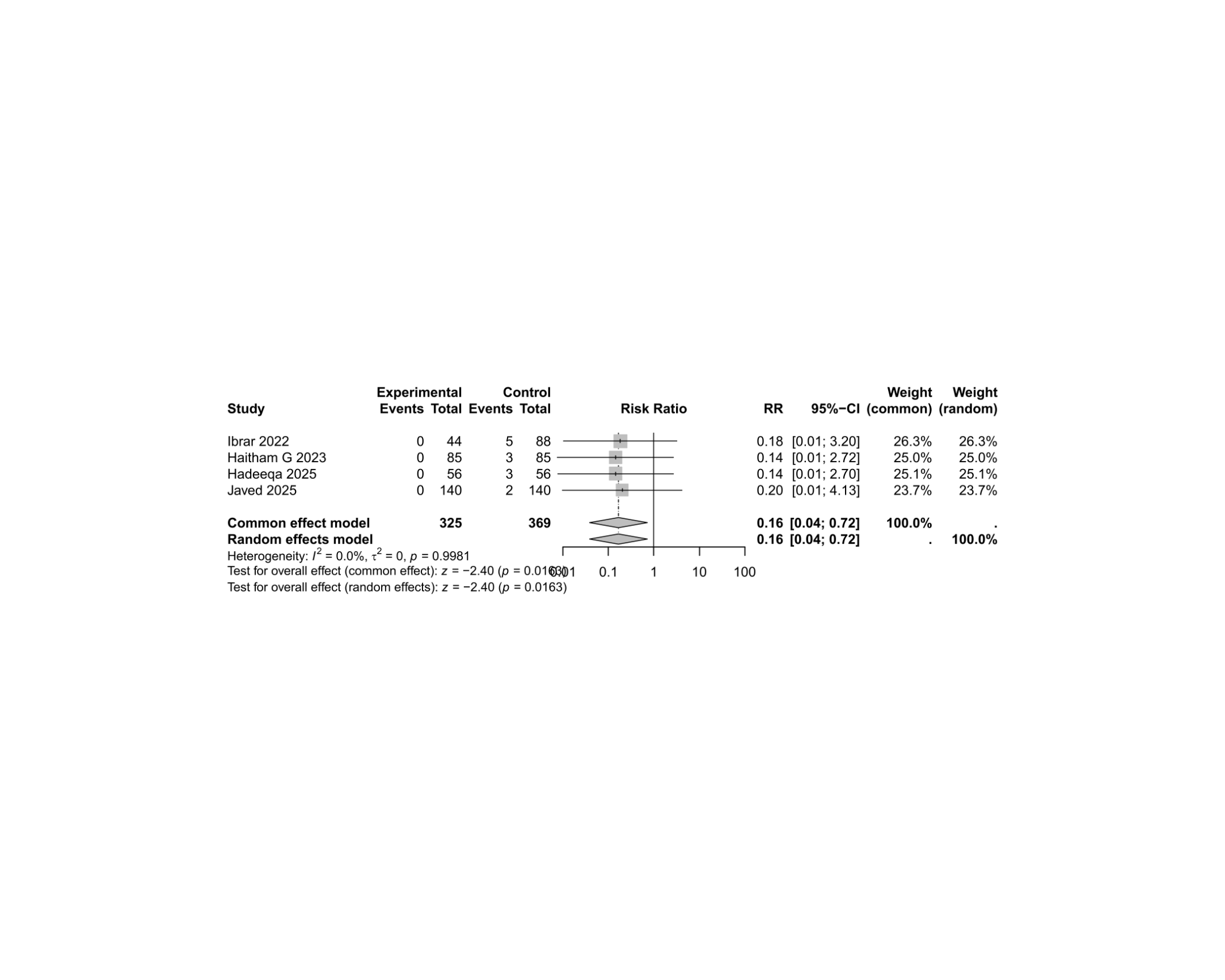


**Supplementary Figure 20:** Funnel plot for assessing publication bias for change in HbA1c.


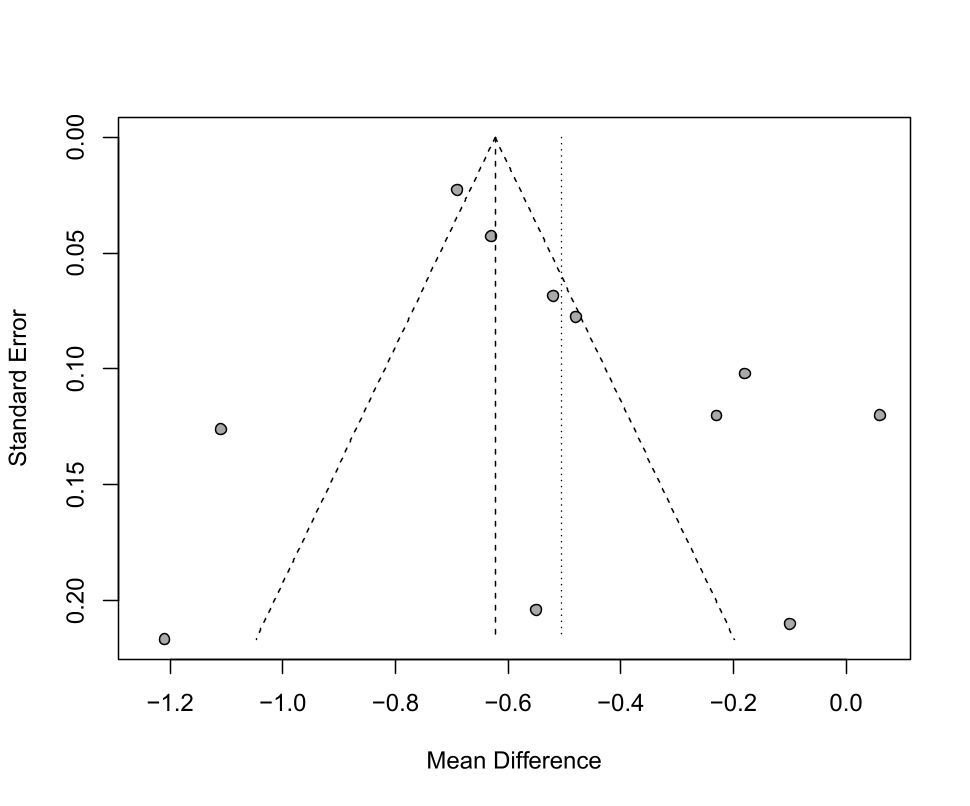


**Supplementary Figure 21:** Funnel plot for assessing publication bias for change in body weight.

**
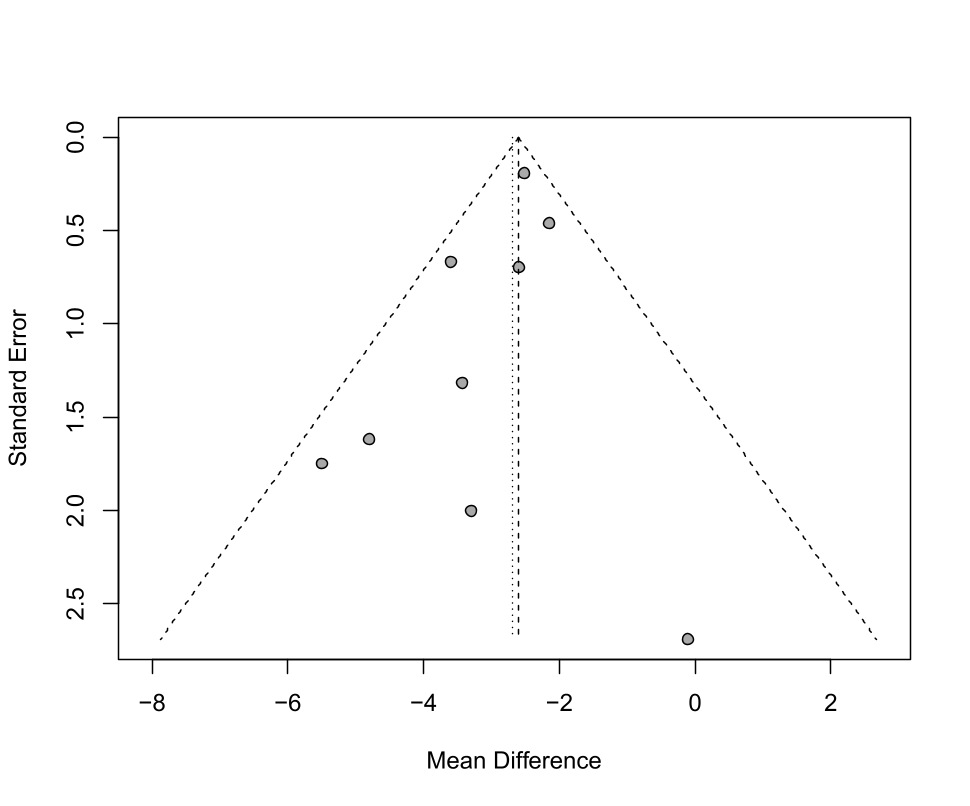
**

**Supplementary Figure 22:** DOI plot and LFK index for assessing publication bias for change in total cholesterol.


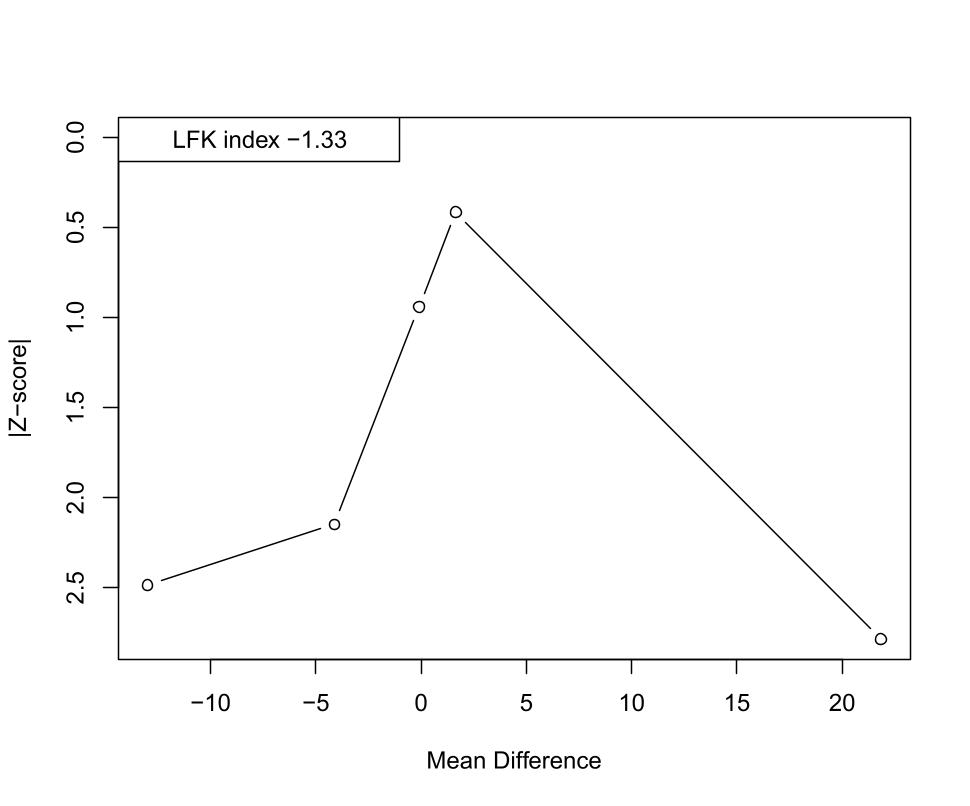


**Supplementary Figure 23:** DOI plot and LFK index for assessing publication bias for change in triglycerides.

**
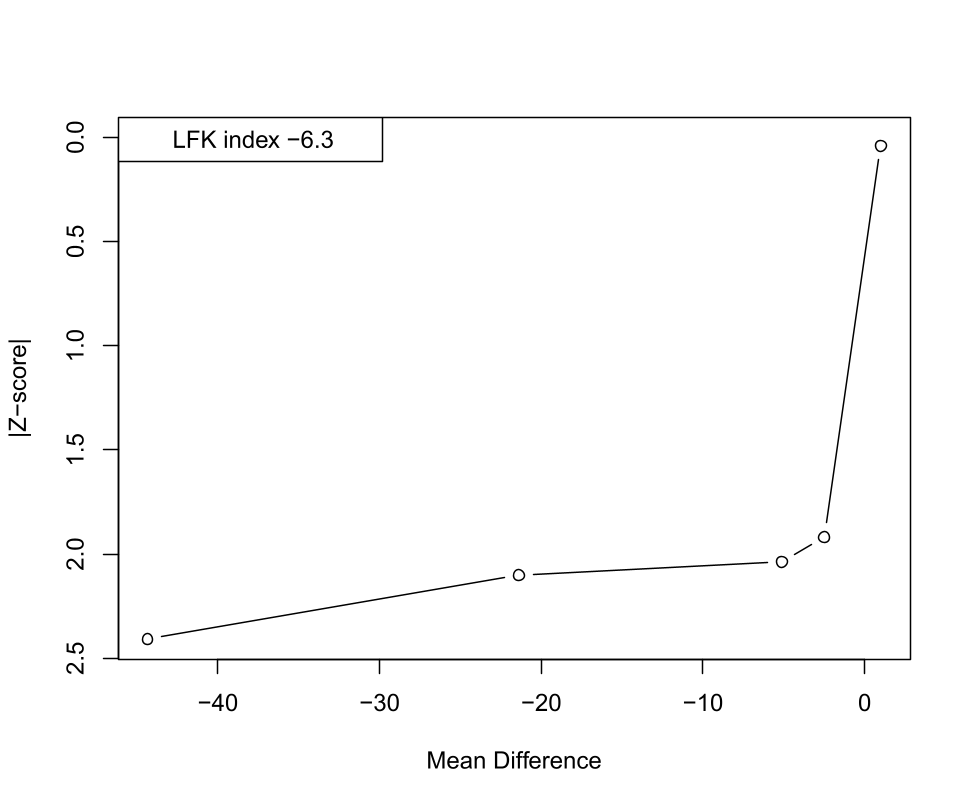
**

**Supplementary Figure 24:** DOI plot and LFK index for assessing publication bias for change in HDL.

**
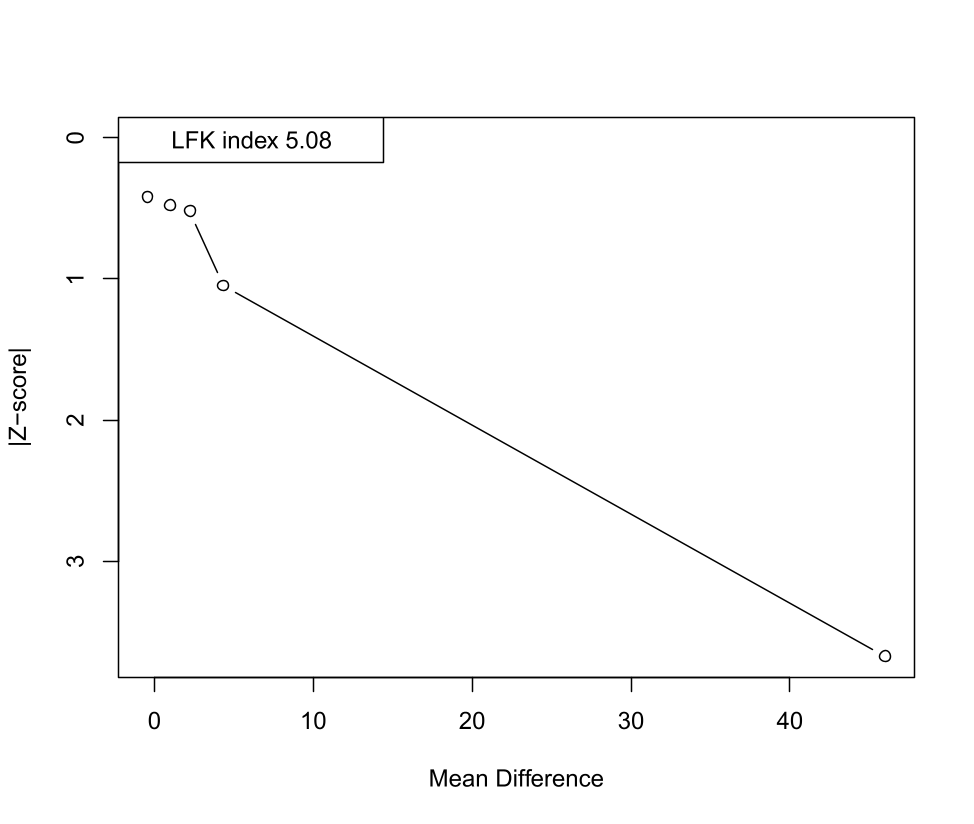
**

**Supplementary Figure 25:** DOI plot and LFK index for assessing publication bias for change in LDL.

**
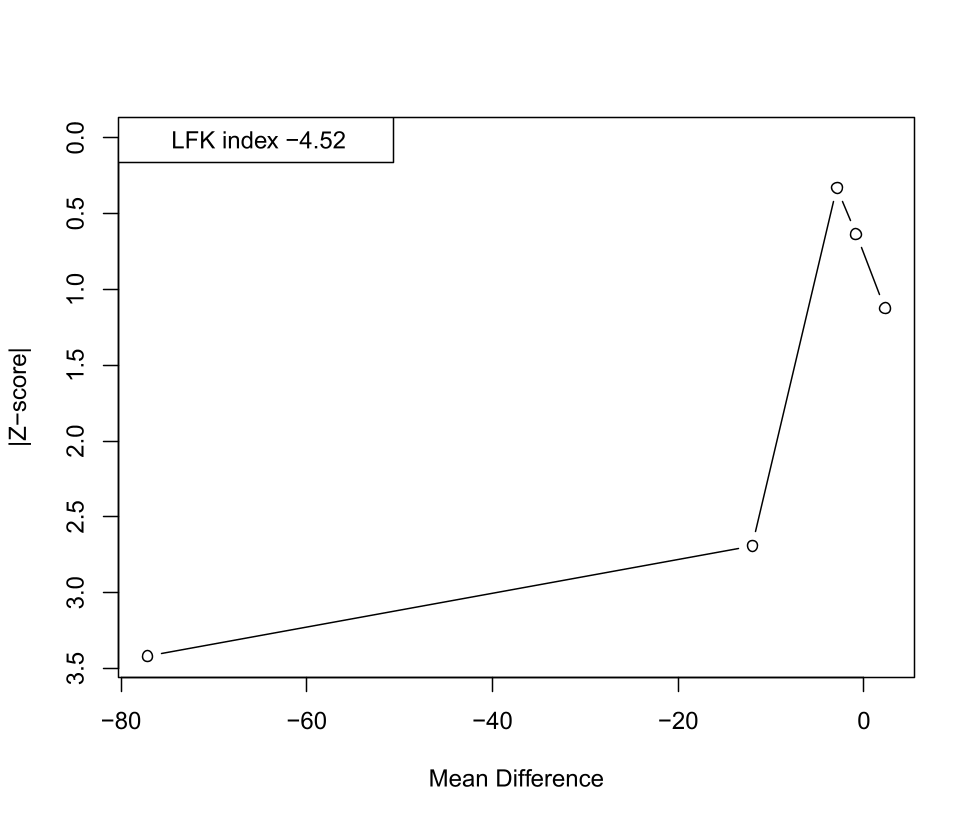
**

**Supplementary Figure 26:** DOI plot and LFK index for assessing publication bias for change in systolic blood pressure.

**
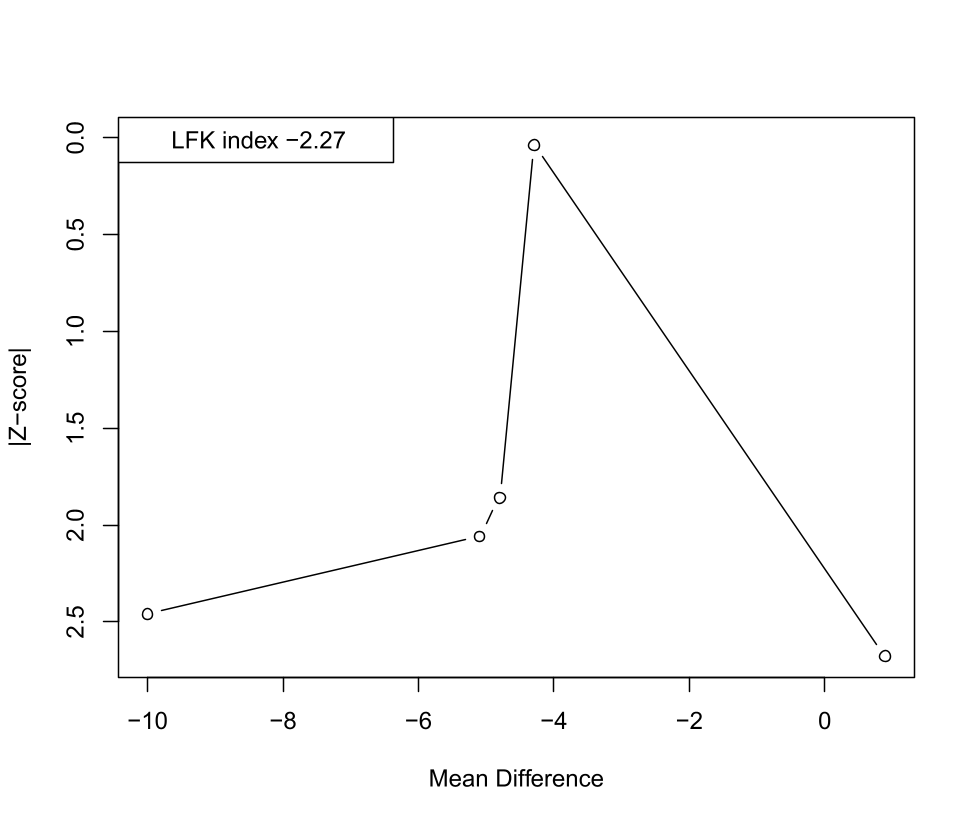
**

**Supplementary Figure 27:** DOI plot and LFK index for assessing publication bias for change in diastolic blood pressure.

**
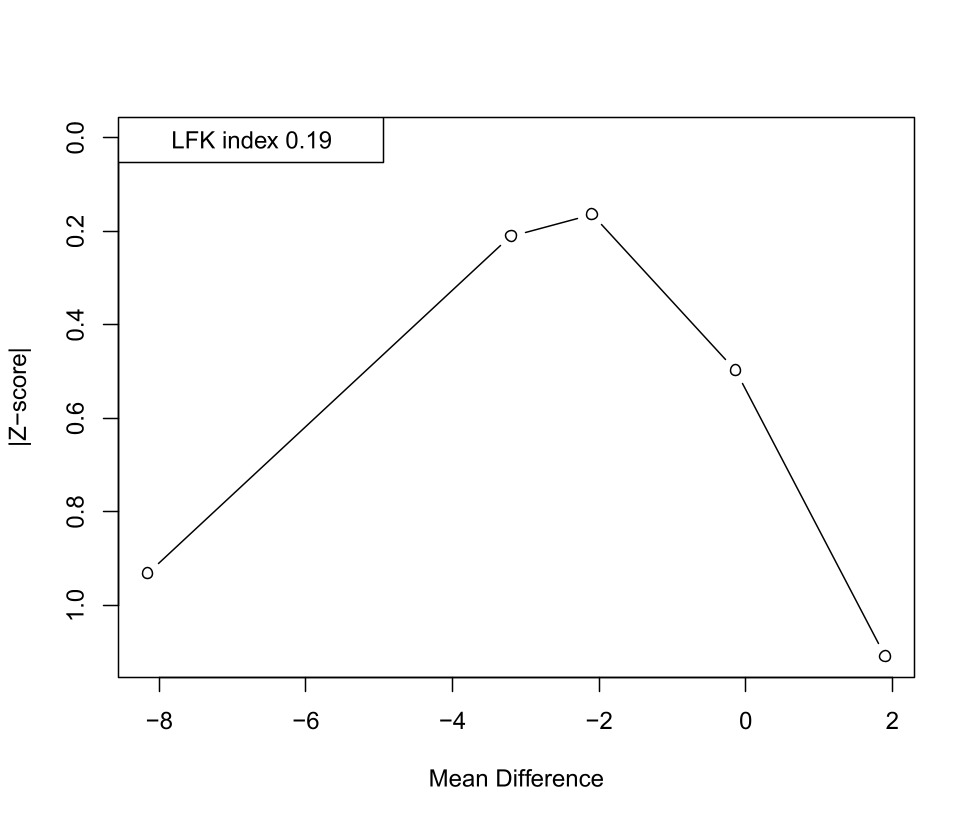
**

**Supplementary Figure 28:** DOI plot and LFK index for assessing publication bias for change in fasting blood glucose.

**
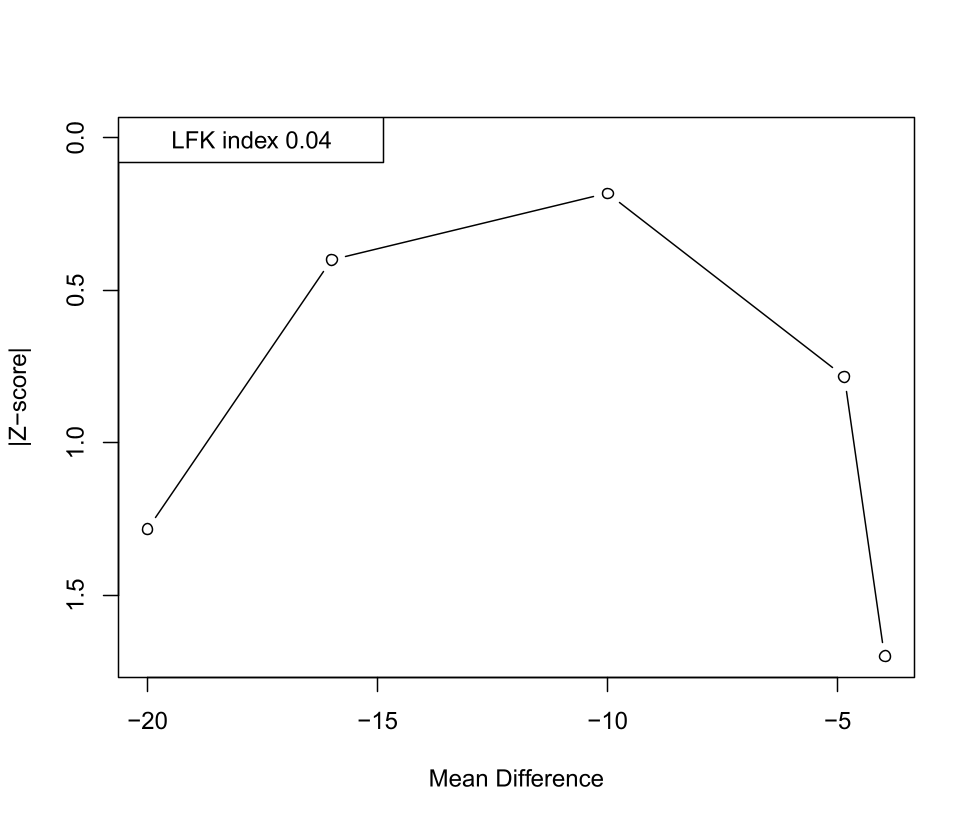
**

**Supplementary Figure 29:** DOI plot and LFK index for assessing publication bias for urinary tract infections.

**
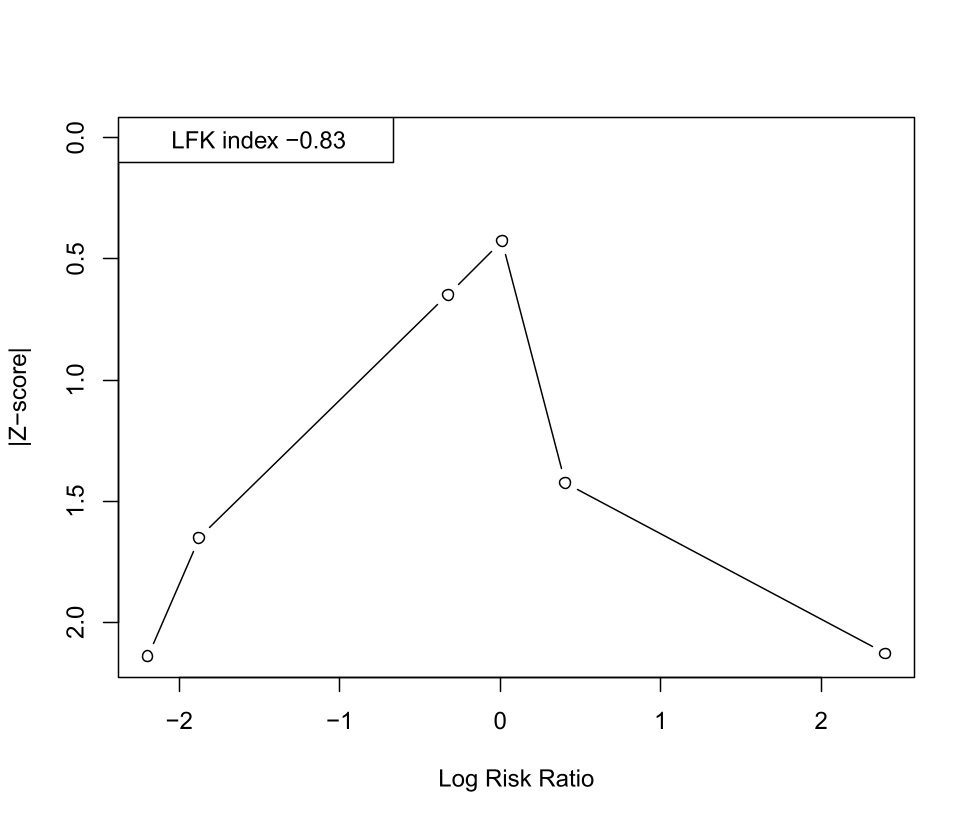
**

**Supplementary Figure 30:** DOI plot and LFK index for assessing publication bias for genital infections.

**
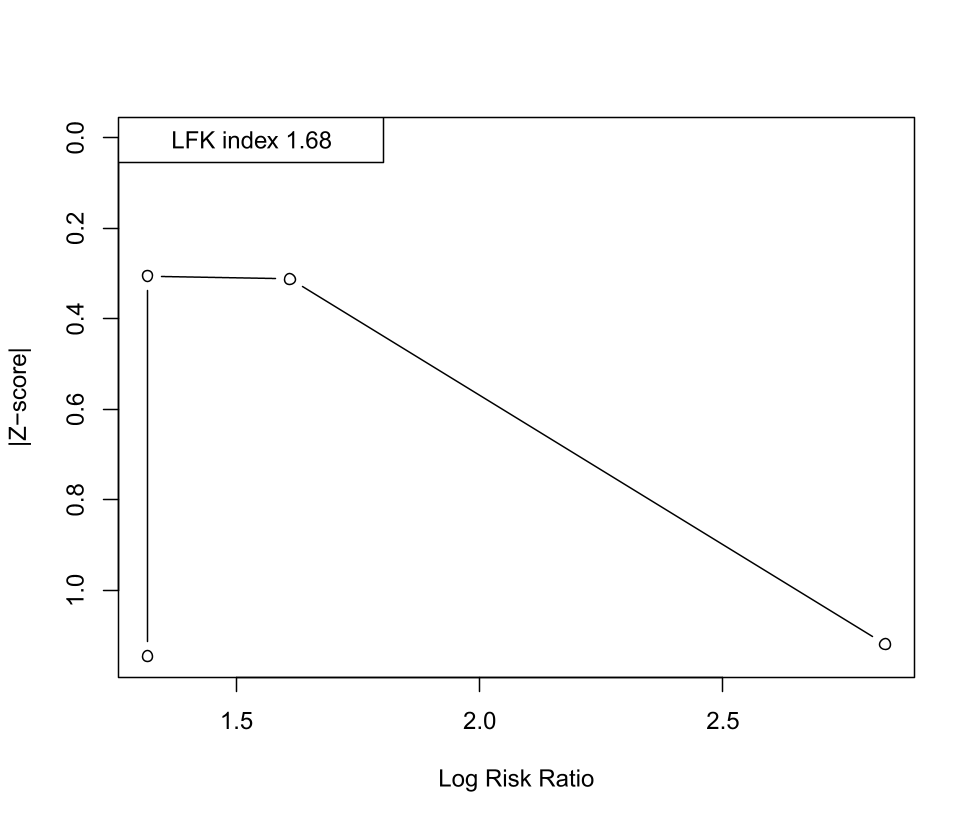
**

**Supplementary Figure 31:** DOI plot and LFK index for assessing publication bias for gastrointestinal disturbances.

**
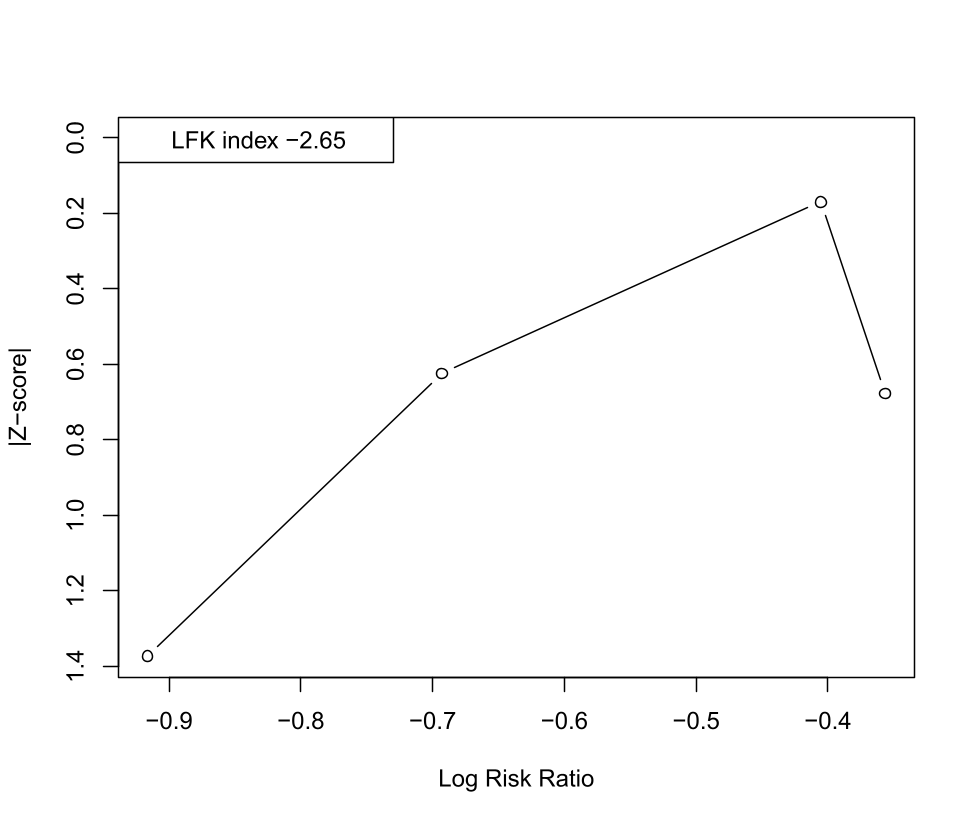
**

**Supplementary Figure 32:** DOI plot and LFK index for assessing publication bias for rash/allergy.


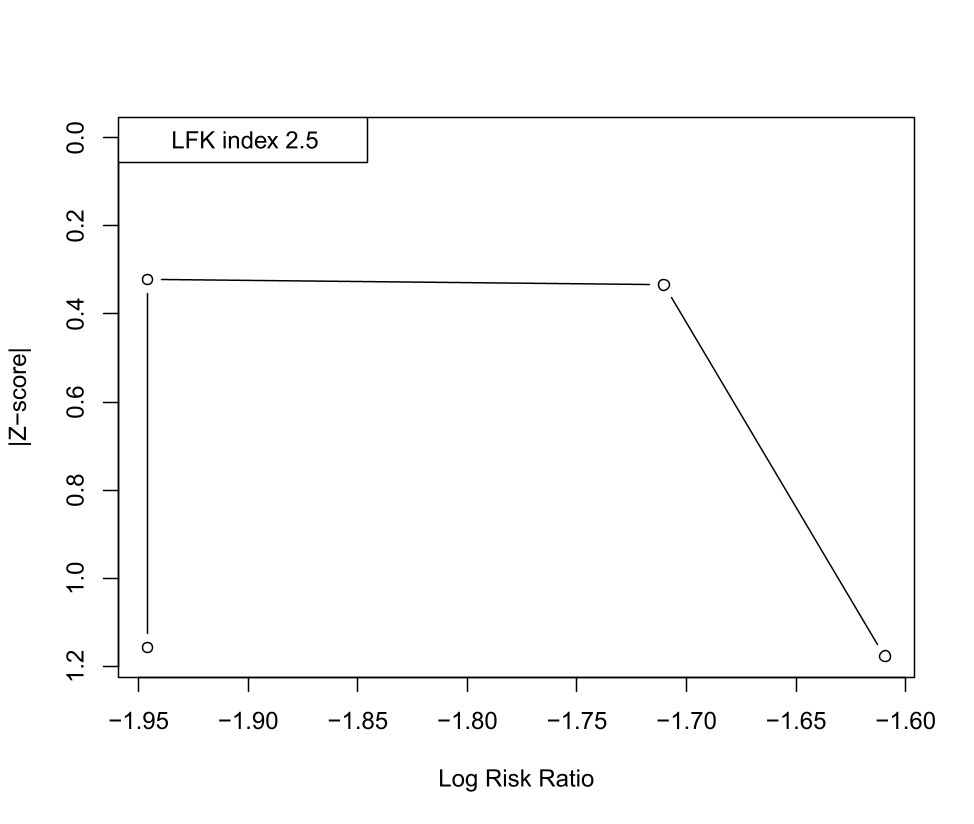


**Supplementary Figure 33:** Meta-regression analysis assessing the association between empagliflozin dose and change in body weight.


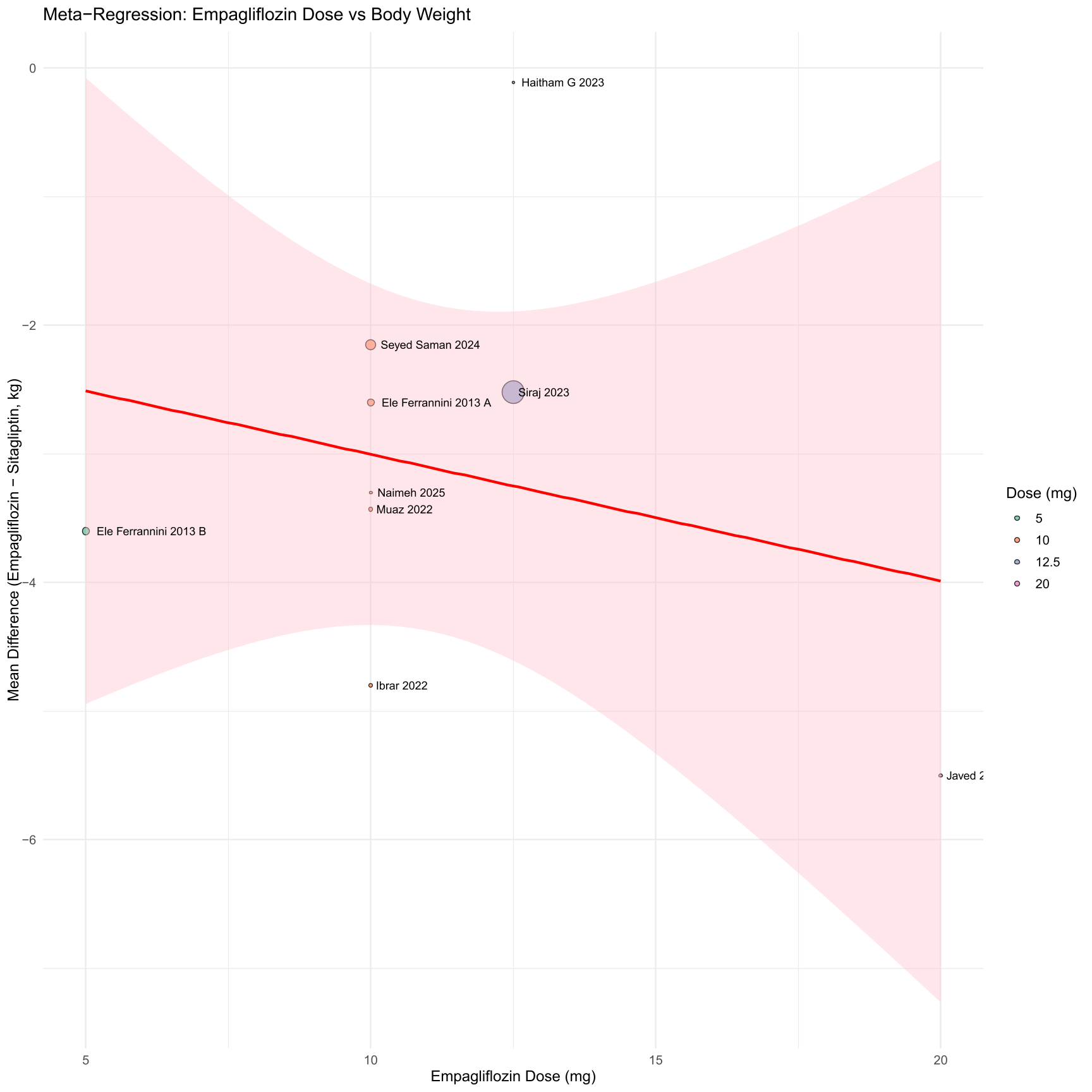


**Supplementary Figure 34:** Meta-regression analysis assessing the association between empagliflozin dose and change in HbA1c.


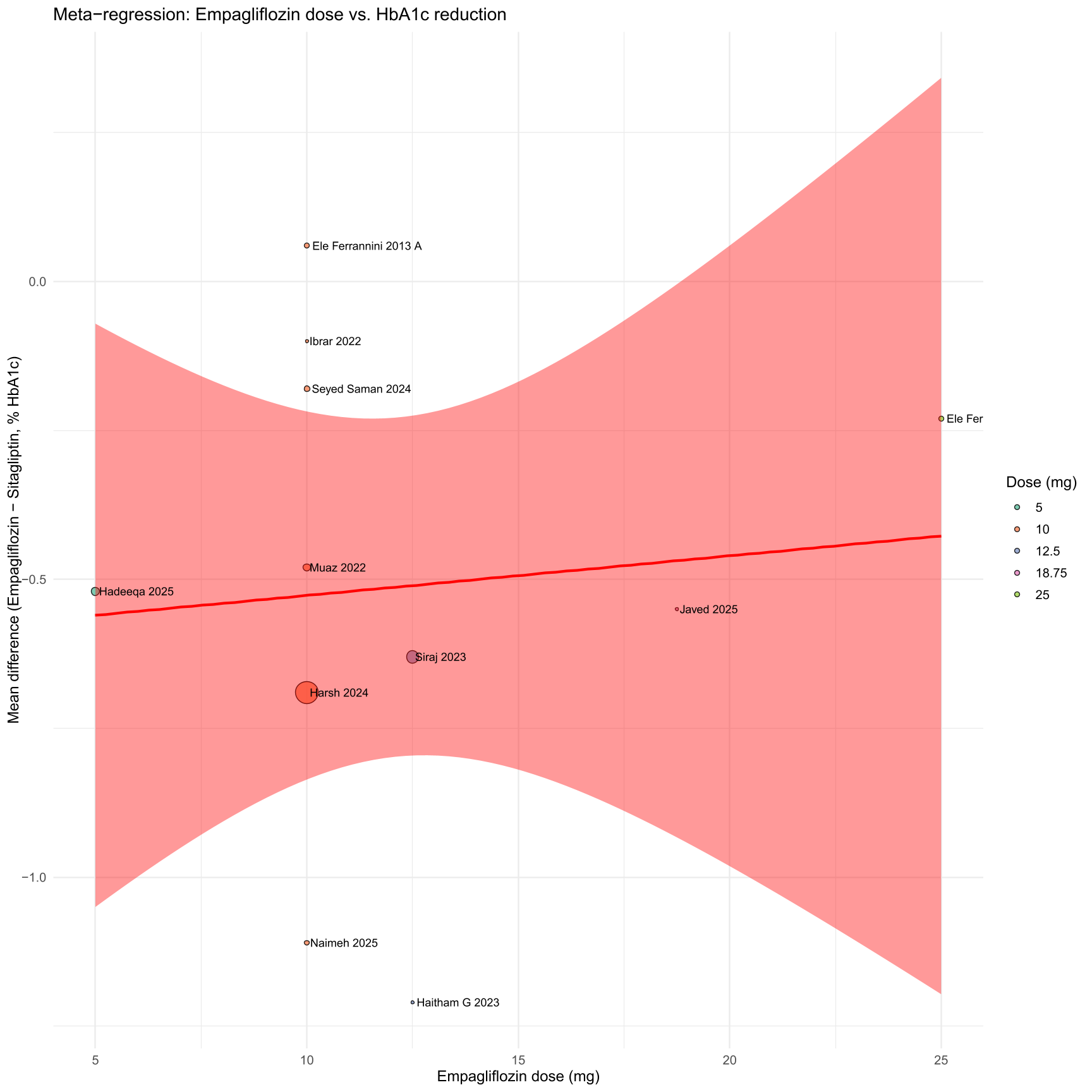

Supplement: Supplementary file 1 — Figure S1: Risk of bias assessment for included randomized controlled trials. Figure S2: Leave‐one‐out sensitivity analysis for change in HbA1c. Figure S3: Leave‐one‐out sensitivity analysis for change in total cholesterol. Figure S4: Leave‐one‐out sensitivity analysis for change in triglycerides. Figure S5: Forest plot for change in HDL comparing empagliflozin + metformin versus sitagliptin + metformin. Figure S6: Leave‐one‐out sensitivity analysis for change in HDL. Figure S7: Forest plot for change in LDL comparing empagliflozin + metformin versus sitagliptin + metformin. Figure S8: Leave‐one‐out sensitivity analysis for change in LDL. Figure S9: Forest plot for change in systolic blood pressure comparing empagliflozin + metformin versus sitagliptin + metformin. Figure S10: Leave‐one‐out sensitivity analysis for change in systolic blood pressure. Figure S11: Forest plot for change in diastolic blood pressure comparing empagliflozin + metformin versus sitagliptin + metformin. Figure S12: Leave‐one‐out sensitivity analysis for change in diastolic blood pressure. Figure S13: Forest plot for change in fasting blood glucose comparing empagliflozin + metformin versus sitagliptin + metformin. Figure S14: Leave‐one‐out sensitivity analysis for change in fasting blood glucose. Figure S15: Forest plot for urinary tract infections comparing empagliflozin + metformin versus sitagliptin + metformin. Figure S16: Leave‐one‐out sensitivity analysis for urinary tract infections. Figure S17: Forest plot for genital infections comparing empagliflozin + metformin versus sitagliptin + metformin. Figure S18: Forest plot for gastrointestinal disturbances comparing empagliflozin + metformin versus sitagliptin + metformin. Figure S19: Forest plot for rash/allergy comparing empagliflozin + metformin versus sitagliptin + metformin. Figure S20: Funnel plot for assessing publication bias for change in HbA1c. Figure S21: Funnel plot for assessing publication bias for change in body weight. Fi [file EDM2-9-e70238-s001.docx]
